# Mechanism of Action of MAO’s *Molecular Cousin*

**DOI:** 10.1021/acscatal.5c05698

**Published:** 2025-10-10

**Authors:** Gaia Urciuoli, Francesco Zaccaria, Cristiano Zuccaccia, Roberta Cipullo, Peter H. M. Budzelaar, Gabriel Menendez Rodriguez, Leonardo Tensi, Antonio Vittoria, Christian Ehm, Vincenzo Busico, Alceo Macchioni

**Affiliations:** † Department of Chemical Sciences, 9307 Federico II University of Naples, Napoli 80126, Italy; ‡ Department of Chemistry, Biology and Biotechnology, 9309 University of Perugia and CIRCC, Perugia 06123, Italy; § DPI, P.O. Box 902, 5600 AX Eindhoven, the Netherlands; ∥ Department of Pharmaceutical Sciences, University of Perugia, Via Del Liceo 1, Perugia 06123, Italy

**Keywords:** catalyst activation, impurity scavenging, methylaluminoxane, borates, polymerization, heterodinuclear adducts

## Abstract

The aluminum-alkyl borate (AAB) salt {[*i*Bu_2_(DMA)­Al]_2_(μ-H)}^+^[B­(C_6_F_5_)_4_]^−^ (**AlHAl_DMA**; DMA = *N,N*-dimethylaniline) is able of fully activating dichloride precatalysts for olefin polymerization and serving as an impurity scavenger, thus deserving to be called a *molecular cousin* of the well-established methylaluminoxane (MAO). With respect to MAO, it offers the advantage of having a well-defined molecular structure, which was exploited herein to investigate its mechanism of action as a cocatalyst. Particularly, the reaction of the precatalyst (Me_2_SiCp_2_)­ZrCl_2_ with **AlHAl_DMA** and with stable [Al*i*Bu_2_(L)]^+^, modeling the putative abstracting species [Al*i*Bu_2_(DMA)]^+^, was studied. The latter reaction led to the isolation of a rare, singly bridged Zr–(μ-Cl)–Al heterodinuclear adduct (**2**), which is a plausible intermediate of chloride abstraction from the precatalyst. Addition of di-*iso*-butylaluminum hydride (DIBAL-H) to **2** yielded a mixture of several multinuclear Zr/Al adducts with bridging μ-Cl and μ-H fragments (**3–6**), which were fully characterized by in-depth 2D NMR spectroscopy. Analogous products were observed in the reaction between (Me_2_SiCp_2_)­ZrCl_2_ and **AlHAl_DMA**, reinforcing the hypothesis that they are intermediates of chloride/hydride exchange, which generates a polymerization-active Zr–H species. The solid-state structure of [(Me_2_SiCp_2_)­Zr]_2_(μ-H)­(μ-Cl)­(μ^2^ -*i*Bu_2_AlH_2_) (**5**) was determined by single-crystal X-ray diffraction. The presence of the μ-H fragment in **AlHAl_DMA** appears to be relevant also for determining the excellent impurity scavenging properties of this cocatalyst, as it was found to react more rapidly than Al–*i*Bu moieties upon exposure of solutions of this cocatalyst to atmospheric oxygen and moisture.

## Graphical abstract

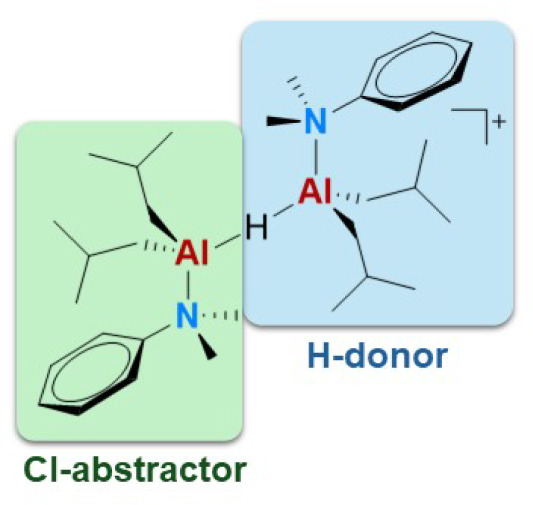

## Introduction

Methylaluminoxane (MAO), the product of the controlled hydrolysis of trimethylaluminum (TMA), is a fascinating compound that has intrigued inorganic and organometallic chemists for decades.^1–3^ Despite its significant commercial importance as a cocatalyst for industrial polyolefin production, its structure and properties remain poorly defined. It is generally accepted that MAO consists of a complex and dynamic distribution of (AlOMe)_*n*_ oligomers and residual TMA from the synthesis.^4–8^ The latter component can be found as a free molecule or associated with the alumoxane oligomers to stabilize some otherwise tricoordinate Al centers. Even though a remarkable crystal structure of an alumoxane sheet has been reported recently,^9^ it remains to be understood to what extent this structure is representative of bulk MAO and, particularly, of its reactive component.^10–15^

The complexity of this system has also hampered a full understanding of its reactivity. Based on polymerization and spectroscopic studies, it is evident that MAO is capable of effectively transforming typical group 4 metal (M) dichloride precatalysts into cationic M-alkyl active species via a series of alkylation and abstraction reactions (Scheme 1).^1–3^ However, it remains unclear which specific MAO component is responsible for these steps. Alkylation could be carried out by free TMA and/or some Al–Me group of the alumoxane oligomers.^16–20^ Conversely, the generally accepted hypothesis is that abstraction is due to transient [AlMe_2_]^+^ cations generated *in situ* by specific Al sites, of which some tentative models have been proposed.^4,21–25^ Only indirect evidence for this reactivity could be gathered by trapping such dimethyl cation in the form of donor-stabilized adducts.^4,24,26^

**Scheme 1:**
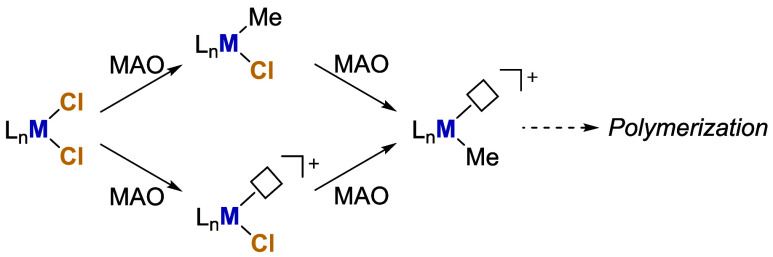
Simplified Activation Mechanism of Dichloride Precatalysts by MAO via a Series of Sequential and/or Concurrent Alkylation and Abstraction Steps[Fn sch1-fn1]

^a^

*M* = group 4 metal; *L* = ancillary ligand.

Interestingly, an analogous transient species, [Al*i*Bu_2_]^+^, is proposed to be responsible for the activation capability of binary cocatalysts, alternative to MAO, which are composed of tri-*iso*-butyl aluminum (TIBAL) and borate salts of Lewis or Brønsted acids like [Ph_3_C]^+^[B­(C_6_F_5_)_4_]^−^ (trityl borate, TTB) or [PhMe_2_NH]^+^ [B­(C_6_F_5_)_4_]^−^ (anilinium borate, AB).^27–29^ This suggests that cationic Al-alkyl species are key in the activation chemistry of molecular olefin polymerization catalysts.

In an attempt to investigate the role of dialkyl cations,^30^ we recently isolated the borate salt of an unusual homodinuclear aluminum-alkyl cation, {[*i*Bu_2_(DMA)­Al]_2_(μ-H)}^+^[B­(C_6_F_5_)_4_]^−^ (**AlHAl_DMA**; DMA = *N,N*-dimethylaniline; Figure 1a).^31^ Remarkably, this species behaves as a complete cocatalyst like MAO, serving as an efficient abstractor and alkylator for typical precatalysts and also as an impurity scavenger to preserve the integrity of the catalytically active species.^31^ Its performance in combination with both metallocenes and “post-metallocenes” is comparable to that of established cocatalysts, even at catalyst concentrations down to 10^–7^ mol/L.^31–34^ With respect to MAO, **AlHAl_DMA** offers the advantage of being a well-defined molecule, which can be easily synthesized and manipulated at room temperature under an inert atmosphere. Furthermore, it is amenable to structural modifications for property modulation, as demonstrated by synthesizing and testing 30 homologues differing for the nature of the neutral ligands coordinated at the Al centers.^35,36^ Even though the prototypical **AlHAl_DMA** remains the best-performing cocatalyst of this class to date, this systematic investigation led to the identification of several interesting Al-alkyl borate (AAB) salts. These include rare examples of stable tricoordinate cationic species, which could be isolated when using bulkier and more shielding ligands than DMA (Figure 1b; *vide infra*).^35,36^

**Figure 1:**
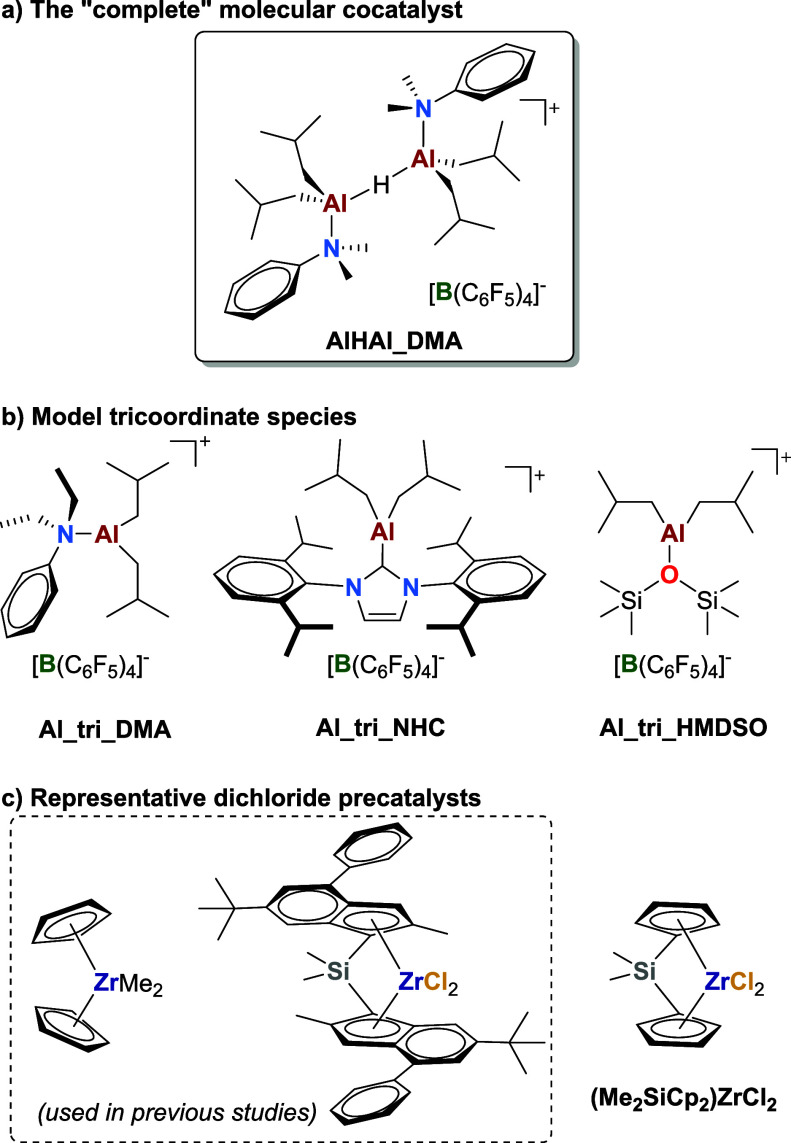
Molecular structure of a) **AlHAl_DMA**, b) model tricoordinate AAB salts for the putative [Al*i*Bu_2_(DMA)]^+^ reactive cation, and c) representative group 4 metal precatalysts.

Another potential advantage deriving from the molecular nature of **AlHAl_DMA** lies in the possibility of using it for mechanistic investigations that are instead prohibitive with MAO. In fact, its organic analogs, TTB and AB, have been crucial for spectroscopic studies of catalytically active species in olefin polymerization, even though they are limited by their ability to serve only as alkyl abstractors.^3,37^ Conversely, the new cocatalysts can fully activate dialkyl and dichloride precatalysts also in the absence of TIBAL, working with nearly equimolar amounts with respect to the transition metal.^31^

Thus, we decided to exploit this feature to investigate the reactivity of **AlHAl_DMA** at a molecular level, focusing on both its activating and impurity-scavenging abilities. In a previous report,^31^ we hypothesized that this new cocatalyst is capable of abstracting the first chloride from the precatalyst by the release of an unsaturated and strongly Lewis acidic [Al*i*Bu_2_(DMA)]^+^ fragment (Scheme 2a,b) and exchange of the second chloride with the hydride (Scheme 2c–e) or an *iso*-butyl group (Scheme 2c′–e′) of the remaining Al*i*Bu_2_(DMA)H subunit. However, polymerization studies do not allow one to draw definitive conclusions on the activation mechanism, as a variety of possible reactions can generate the same fragments deriving from insertion into Zr–H and Zr–iBu groups. Furthermore, preliminary spectroscopic studies allowed the observation of only methyl abstraction from the simplest zirconocene, Cp_2_ZrMe_2_ (Figure 1c). Attempts to isolate relevant reaction intermediates with a *bis*-indenyl *ansa*-zirconocene dichloride (Figure 1c) led to complex and dynamic mixtures of unknown products, hampering any definitive conclusions on chloride abstraction and exchange capability.^31^

**Scheme 2:**
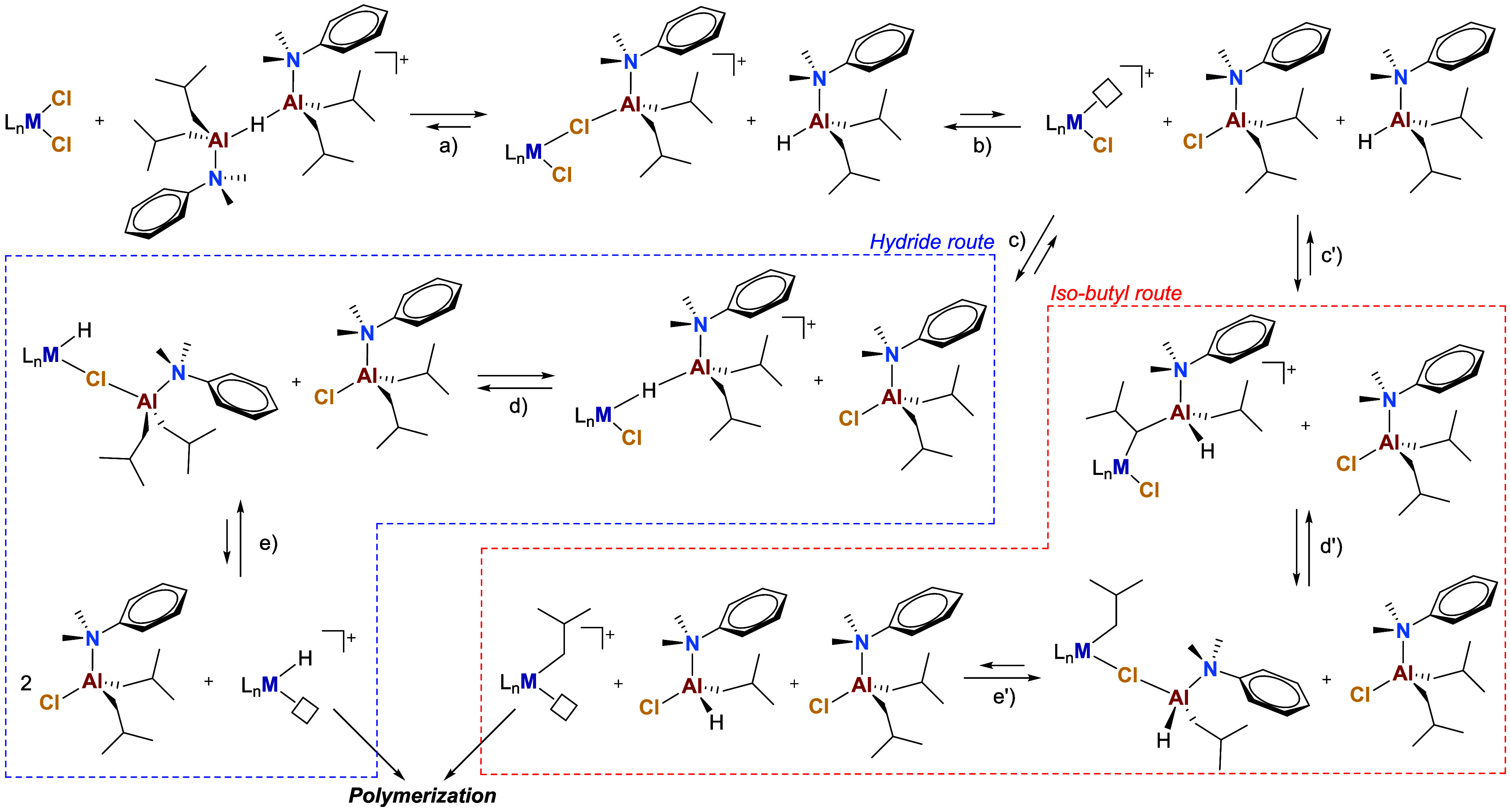
Possible Activation Mechanism of Dichloride Precatalysts by **AlHAl_DMA**, Involving Abstraction of the First Chloride by the [Al*i*Bu_2_(DMA)]^+^ Fragment (a,b) and Exchange of the Second Chloride with a Hydride (c–e) or *iso*-Butyl Group (c′–e′) of the Remaining Al*i*Bu_2_(DMA)H Subunit

Herein, we report an extensive NMR spectroscopic study of the reactivity of **AlHAl_DMA**. Leveraging on experience, we selected (Me_2_SiCp_2_)­ZrCl_2_ as representative precatalyst for activation studies, since it combines the desirable simplicity of the previously used *bis*-Cp catalyst with the rigid ligand backbone and dichloride nature of the *bis*-indenyl one (Figure 1c). Furthermore, [Al*i*Bu_2_(L)]^+^ cations with *L* = *N,N*-diethylaniline (**DEA**), hexamethyldisiloxane (**HMDSO**), and *N*-heterocyclic carbene (**NHC**; Figure 1b)^35,36^ were exploited as suitable models of the putative abstracting species [Al*i*Bu_2_(DMA)]^+^ (Scheme 2a,b). The isolated reaction intermediates provided insights into the roles of the two subunits of **AlHAl_DMA**.

## Results and Discussion

### Chloride Abstraction by Mononuclear AAB Salts

The activating ability of **AlHAl_DMA** was investigated by starting with the chloride abstraction step. For simplicity, the reaction of (Me_2_SiCp_2_)­ZrCl_2_ with an equimolar amount of tricoordinate AAB salts was studied first (Scheme 3). In the case of **Al_tri_HMDSO**, full conversion of the reagents was immediately observed, leading to a stable mixture of products. The latter showed sharp NMR resonances also at 298 K, which 2D NMR experiments allowed to identify as Al-*i*Bu, HMDSO, and Me_2_SiCp_2_Zr groups due to their diagnostic δ­(^1^H) and δ­(^13^C);^28,38–40^ a single set of NMR signals was observed for each of these groups (Figures S1 and S2). ^1^H ROESY NMR experiments showed dipolar interactions between the Al-*i*Bu and Zr-Cp resonances, while no such interactions were observed between these two fragments and the methyl groups of **HMDSO** (Figure S3). The latter appears to diffuse as a free molecule based on ^1^H PGSE NMR (Figure S4).^41,42^ These results therefore point to the formation of a Zr/Al heterodinuclear adduct with two bridging chlorides (**1**; Scheme 3a), resulting from the detachment of **HMDSO** from the [Al*i*Bu_2_]^+^ cation, which then binds to the dichloride metallocene.

**Scheme 3:**
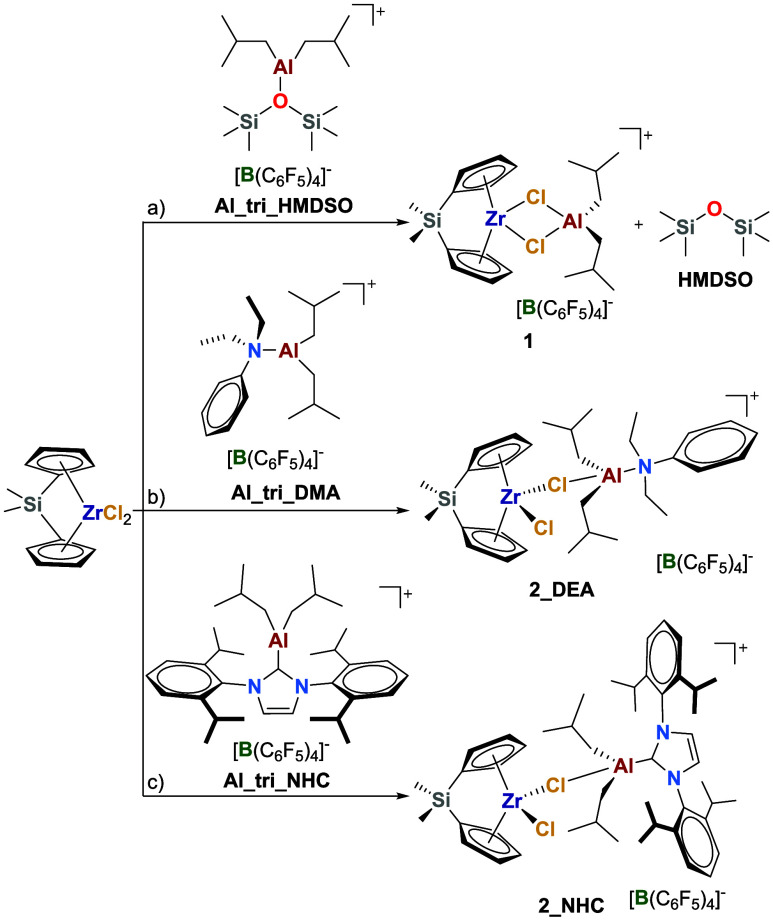
Reaction of (Me_2_SiCp_2_)­ZrCl_2_ with Tricoordinate AAB: a) **Al_tri_HMDSO**, b) **Al_tri_DEA**, c) **Al_tri_NHC**

Despite the strong oxophilicity of the Zr and Al complexes, the weakly basic siloxane ligand^43^ is released and a tetracoordinate donor-stabilized Al center is not observed;^36^ instead the doubly bridged adduct **1** is formed. This kind of adduct has been often observed in activation experiments with traditional cocatalysts, including MAO with its free TMA component,^7,16,44–51^ and it has been proposed to represent potential dormant species in polymerization.^7,45,52^

Similarly, in the case of **Al_tri_DEA**, reaction with (Me_2_SiCp_2_)­ZrCl_2_ led to the rapid formation of products, giving a single set of well-resolved NMR signals at 253 K (Figure 2a,b). In contrast to **HMDSO**, ^1^H ROESY NMR experiments revealed clear dipolar interactions of the Al-*i*Bu groups with both Zr-Cp and **DEA** (Figure 2b), suggesting that these three fragments belong to the same molecule. The reaction product was therefore formulated as a singly bridged Zr–(μ-Cl)–Al heterodinuclear adduct (**2_DEA**), in which the Al center binds two *iso*-butyl groups, **DEA** and a bridging chloride (Scheme 3b). This compound likely undergoes a rapid exchange of the [Al*i*Bu_2_(DEA)]^+^ subunit between the two chlorides of Zr, explaining why the Cp and Si–Me protons appear as broad singlets. Further lowering the temperature led to broad NMR signals likely due to incipient precipitation of ionic products.

**Figure 2:**
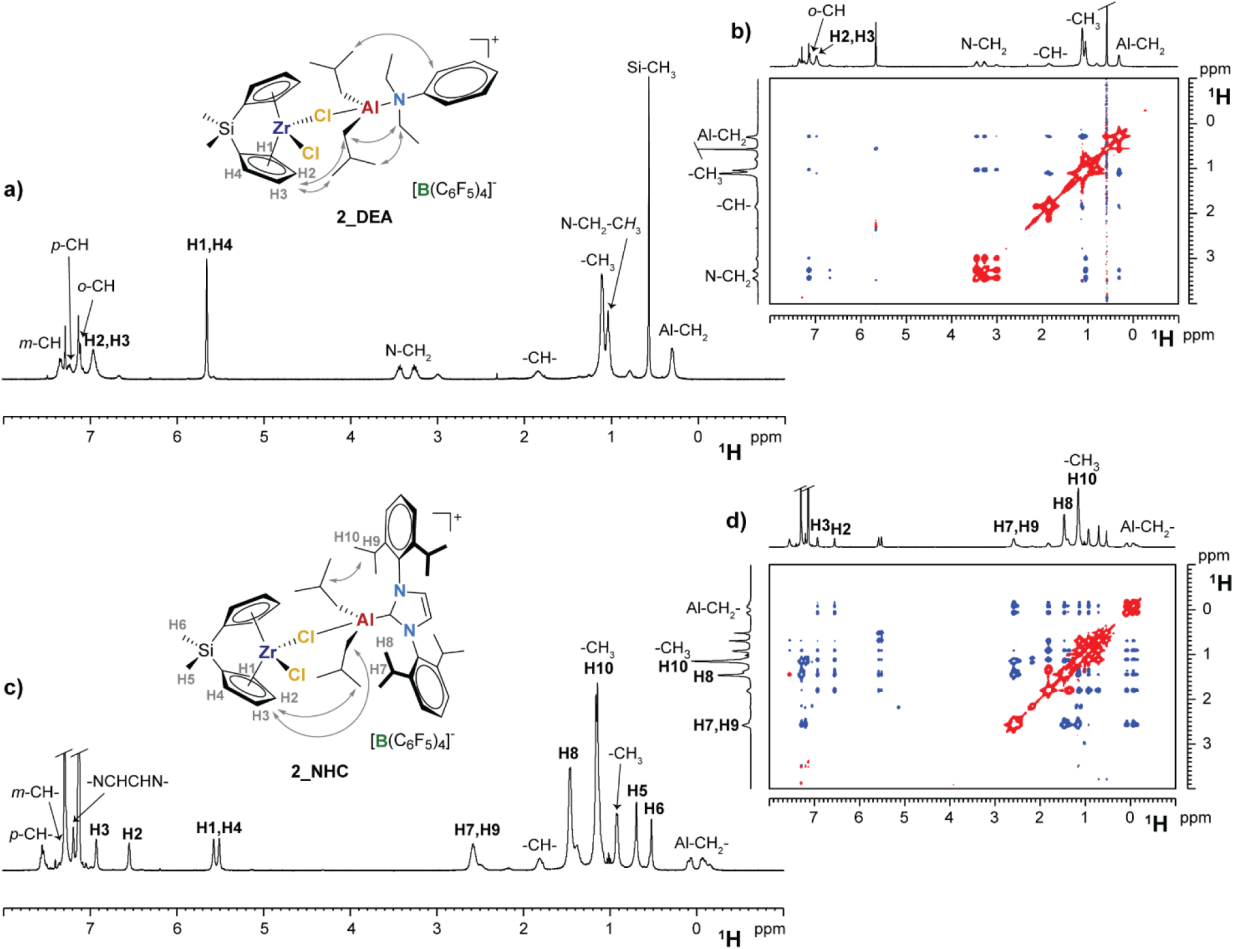
^1^H (a,c) and ^1^H ROESY (b,d) NMR spectra (chlorobenzene-*d*_5_) of **2_DEA** (a,b) and **2_NHC** (c,d) recorded at 253 and 223 K, respectively. Gray arrows highlight relevant dipolar interactions.

Interestingly, analogous results were obtained with **Al_tri_NHC** (Scheme 3c). In this case, at 223 K, the above-described exchange process was slowed down to a point that full characterization of the **2_NHC** adduct was possible. The latter exhibits the expected differentiation of the resonances of the two sides of the (Me_2_SiCp_2_)Zr fragment (Figure 2c,d).

Importantly, these compounds correspond to the intermediate proposed for chloride abstraction by **AlHAl_DMA** (Scheme 2a,b), supporting the hypothesis that the neutral ligand can stabilize [Al*i*Bu_2_(L)]^+^ fragments capable of reacting with dichloride precatalysts. The tricoordinate AAB salts therefore proved to be suitable model compounds, allowing for the trapping of plausible intermediates for chloride abstraction without triggering subsequent chloride exchange reactions. This is also coherent with polymerization experiments showing that mononuclear AAB salts are incapable of fully activating dichloride precatalysts in the absence of alkylation reagents like TIBAL or di-*iso*-butyl aluminum hydride (DIBAL-H).^31,35,36^

Furthermore, while several neutral analogs are known,^53–59^ **2_DEA** and **2_NHC** represent very rare examples of cationic singly bridged Zr/Al heterodinuclear adducts and—to the best of our knowledge—the first ones ever reported in the context of olefin polymerization catalysis. It is worth noting that they can be seen as higher-energy intermediates toward the generation of the active species compared to doubly bridged analogs like **1**, consistent with the fact that they could only be manipulated at low temperatures. They can therefore prove useful also in the broader context of mechanistic investigations of olefin polymerization, where heterodinuclear adducts are relevant not only for catalyst activation but also for the understanding of deactivation processes,^7,45,52^ chain termination,^60–62^ and coordinative chain transfer polymerization.^63–65^

### Chloride Exchange in the Presence of DIBAL-H

After the abstraction of the first Zr–Cl, the replacement of the second chloride with a group capable of undergoing olefin migratory insertion was investigated. Since **AlHAl_DMA** might exchange both a hydride or an *iso*-butyl group with the precatalysts (Scheme 2), **2_DEA** and **2_NHC** were reacted with DIBAL-H aiming to trap relevant reaction intermediates and discriminate between these two possibilities.

The reaction of **2_DEA** with DIBAL-H resulted in a complex mixture of products that could not be characterized even at a low temperature. Gratifyingly, the reaction with **2_NHC** provided a complex, yet stable, mixture of products showing sharp NMR resonances at 298 K (Figure 3). In-depth 2D NMR characterization allowed the identification of several Zr-Cp and Al-*i*Bu fragments at diagnostic δ­(^1^H) and δ­(^13^C) (Figures 3, S9, and S10).^28,38–40^ Furthermore, several doublets and triplets were observed at very low δ­(^1^H) values between 0 and −7 ppm, which show no scalar correlations with C atoms in the^1^H–^13^C HSQC NMR spectrum (Figure S9). Given their peculiar chemical shifts, they were assigned to Zr–H species.^28,66–70^

**Figure 3:**
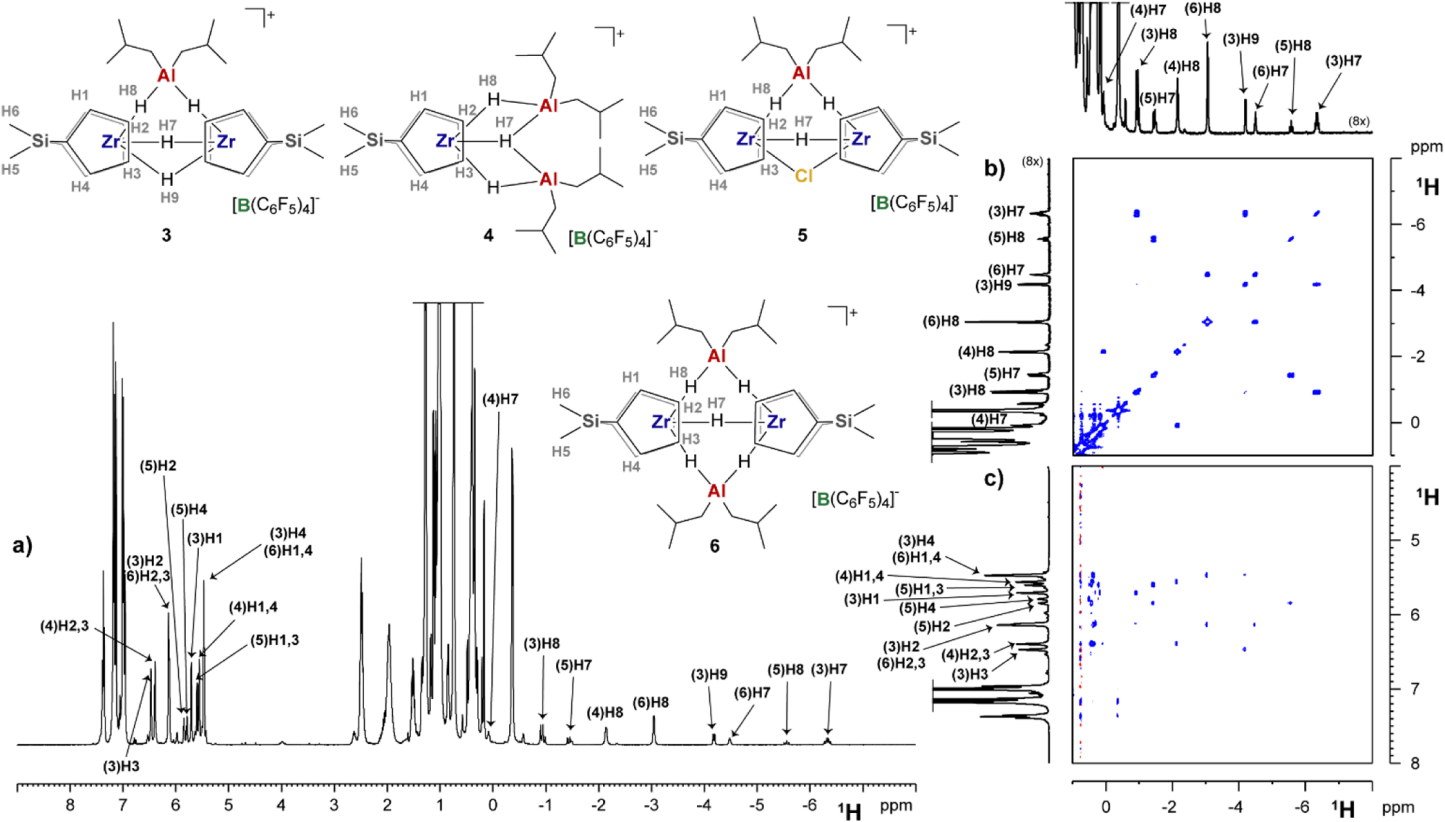
Sections of relevant NMR spectra (chlorobenzene-*d*_5_, 298 K) of the products obtained from the reaction of **2_DEA** with DIBAL-H: a) ^1^H NMR, b) ^1^H COSY NMR, and c) ^1^H NOESY NMR. Assignment of alkyl groups is omitted for clarity (see Supporting Information).

Analysis of the complex set of dipolar interactions detected by ^1^H NOESY NMR (Figures 3c and S10), signal multiplicity, and relative peak integrals allowed to identify all the major reaction products as the variety of multinuclear Zr/Al adducts shown in Scheme 4 (**3–6**; see also Figure 3 and Supporting Information). This interpretation is in line with literature studies,^28,66–68^ demonstrating that formally pentacoordinate Zr centers with bridging Cl or H groups can be obtained starting from simple *bis*-Cp zirconocenes.^69,71–73^ A complex variety of resonances relative to NHC fragments were detected; among them, only those of some **Al_tri_NHC** could be assigned.

**Scheme 4:**
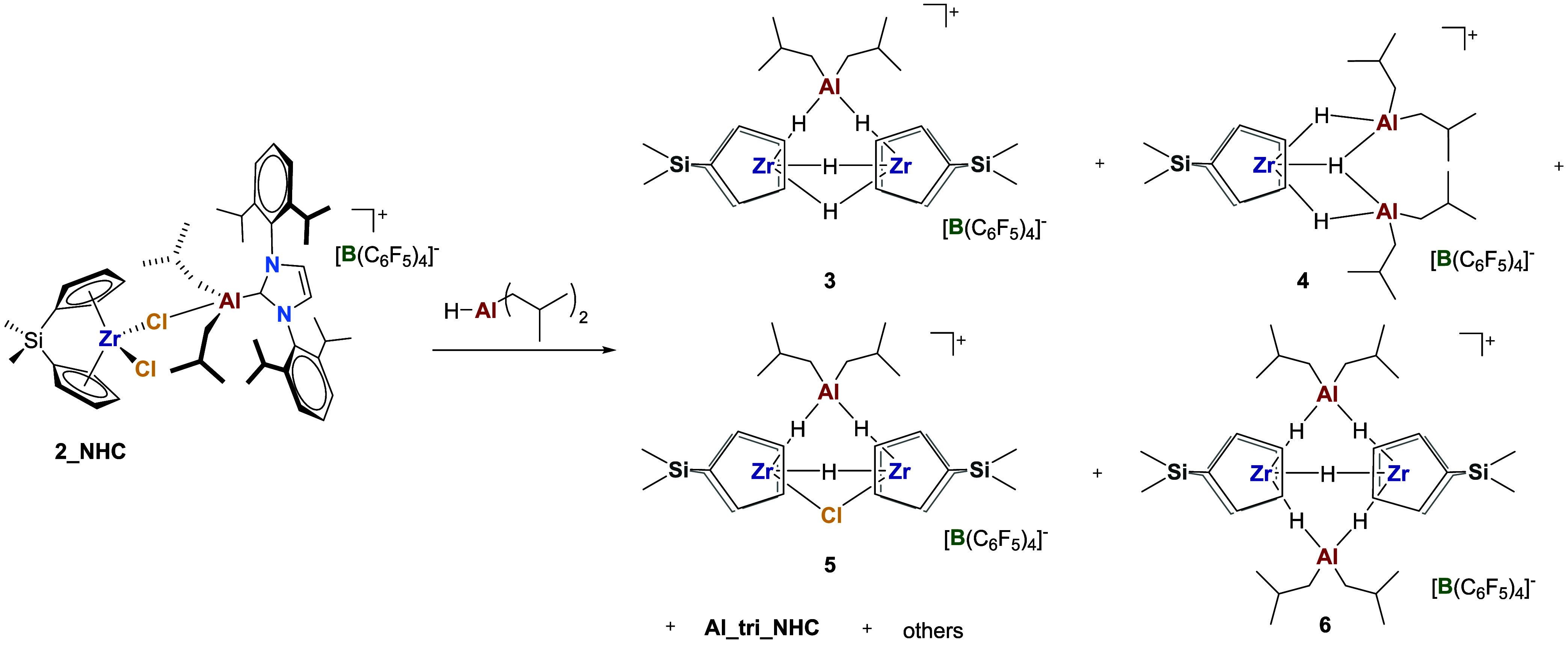
Reaction of 2_NHC with DIBAL-H[Fn sch4-fn1]

^a^
Reaction products are depicted from the top view for simplicity. DIBAL-H is shown as a monomer for simplicity.

Single crystals of mono- and dicationic homodinuclear Zr adducts **7–8** were obtained and their structures are shown in Figure 4a. Nevertheless, no NMR spectroscopic evidence of their presence in solution was obtained. Those species are likely formed upon the slow decomposition of **3–6** during crystallization attempts, as Al–H and Al–C bonds are particularly susceptible to side reactions.

**Figure 4:**
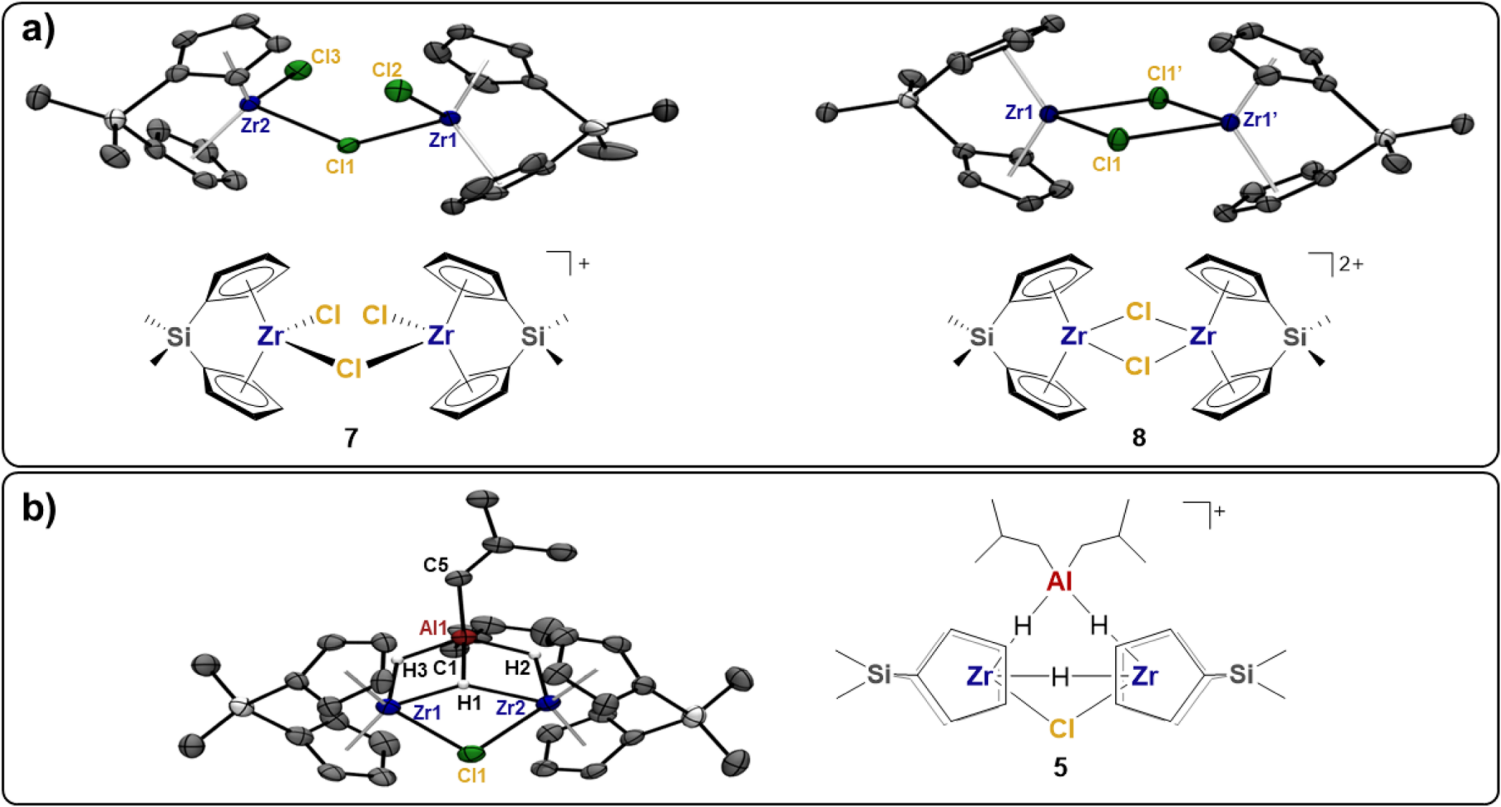
Ortep drawings of crystal structures obtained by single-crystal X-ray diffraction: (a) the species crystallized from the **2_NHC**/DIBAL-H reaction mixture, **7** and **8**; (b) one of the products of the reaction between (Me_2_SiCp_2_)­ZrCl_2_ with **AlHAl_DMA**, namely **5**. Ellipsoids are drawn at the 50% probability level. Hydrogen atoms, the [B­(C_6_F_5_)_4_]^−^ anion, and solvent molecules are omitted for clarity. Relevant distances (Å) and angles (deg) of **7**: Zr1–Cl1 2.5685(8), Zr1–Cl2 2.4037(8), Zr2–Cl3 2.4048(8), Cl2–Zr1–Cl1 93.68(3), Zr1–Cl1–Zr2 132.51(3), Cl3–Zr2–Cl1 92.88(3). Relevant distances (Å) and angles (deg) of **8**: Zr1–Cl1 2.5646(6), Zr1–Cl1′ 2.5642(6), Cl1–Zr1–Cl1′ 84.31(2). Relevant distances (Å) and angles (deg) of **5**: Zr1–H1 2.06(7), Zr2–H1 1.91(7), Zr1–H3 1.89(11), Zr2–H2 1.87(11), Zr1–Cl1 2.5975(17), Cl1–Zr2 2.5963(18), Al1–H1 1.90(7), Al1–H3 1.81(11), Al1–H2 1.79(11), Al1–C5 1.976(7), C1–Al1 1.971(7), Cl1–Zr1–H1 68.(2), Cl1–Zr2–H1 70.(2), Zr2–Cl1–Zr1 89.12(5), H1–Zr2–H2 58.(4), H1–Zr1–H3 62.(4), H1–Al1–H3 66.(4), H1–Al1–H2 60.(4), see Supporting Information for additional details.

With respect to the activation mechanism of **AlHAl_DMA**, it is important to note that all of the isolated Zr species are cationic, indicating that **Al_tri_NHC** has successfully abstracted a Cl from the precatalyst. Furthermore, only μ-Cl and μ-H fragments are formed, while all *iso*-butyl groups remain bound solely to Al. This suggests that a Zr–H (Scheme 2c–e), not a Zr–*i*Bu (Scheme 2c′–e′) is preferentially formed upon precatalyst activation.

### Full Precatalyst Activation with **AlHAl_DMA**

The reaction of (Me_2_SiCp_2_)­ZrCl_2_ with the full activator, **AlHAl_DMA**, led to a complex, yet well-resolved, set of NMR resonances (Figure 5 and Scheme 5). The reagents were initially mixed at 228 K, where NMR spectra clearly showed the formation of **3** and **5** (Figure 5a), already observed in the reaction of **2_NHC** with DIBAL-H. This demonstrates that these compounds are indeed relevant in the activation of dichloride precatalysts by **AlHAl_DMA**.

**Scheme 5:**
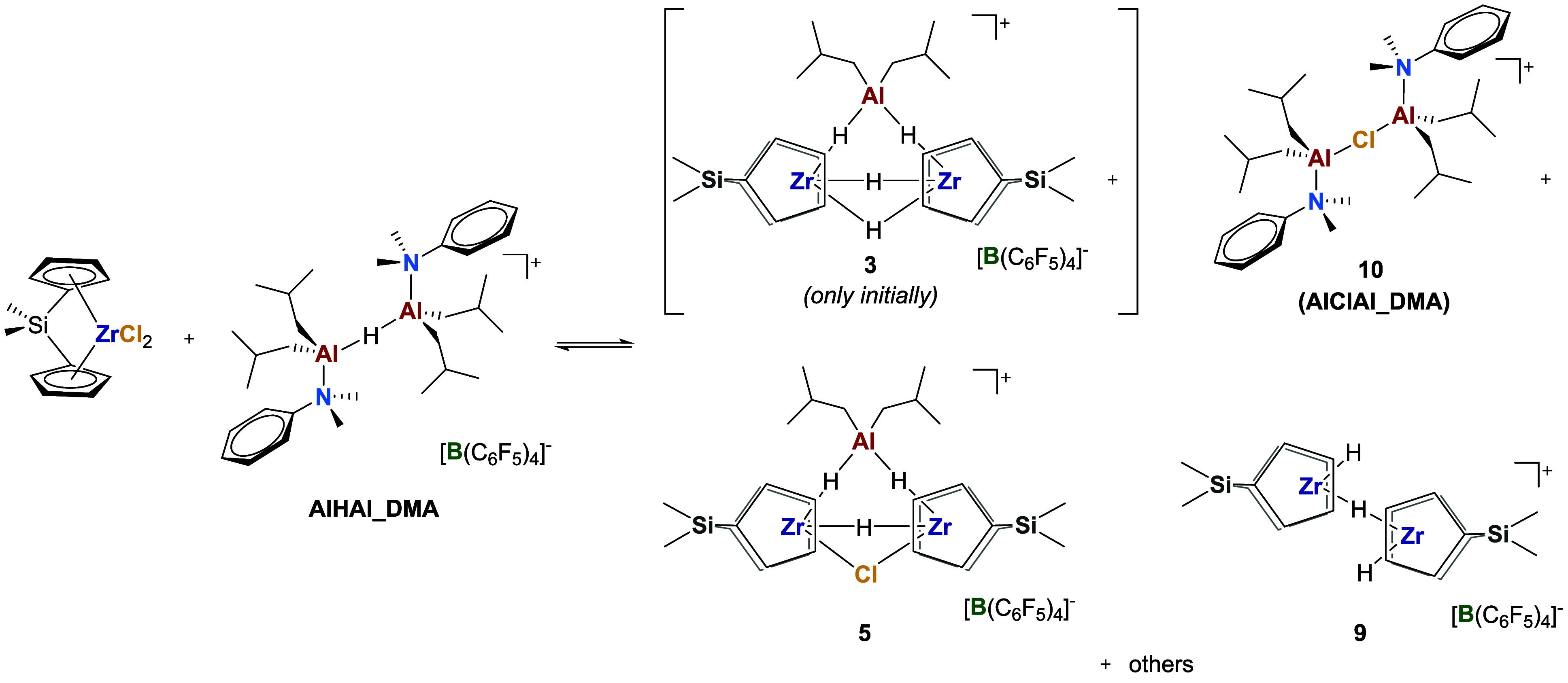
Reaction of (Me_2_SiCp_2_)­ZrCl_2_ with **AlHAl_DMA**

**Figure 5:**
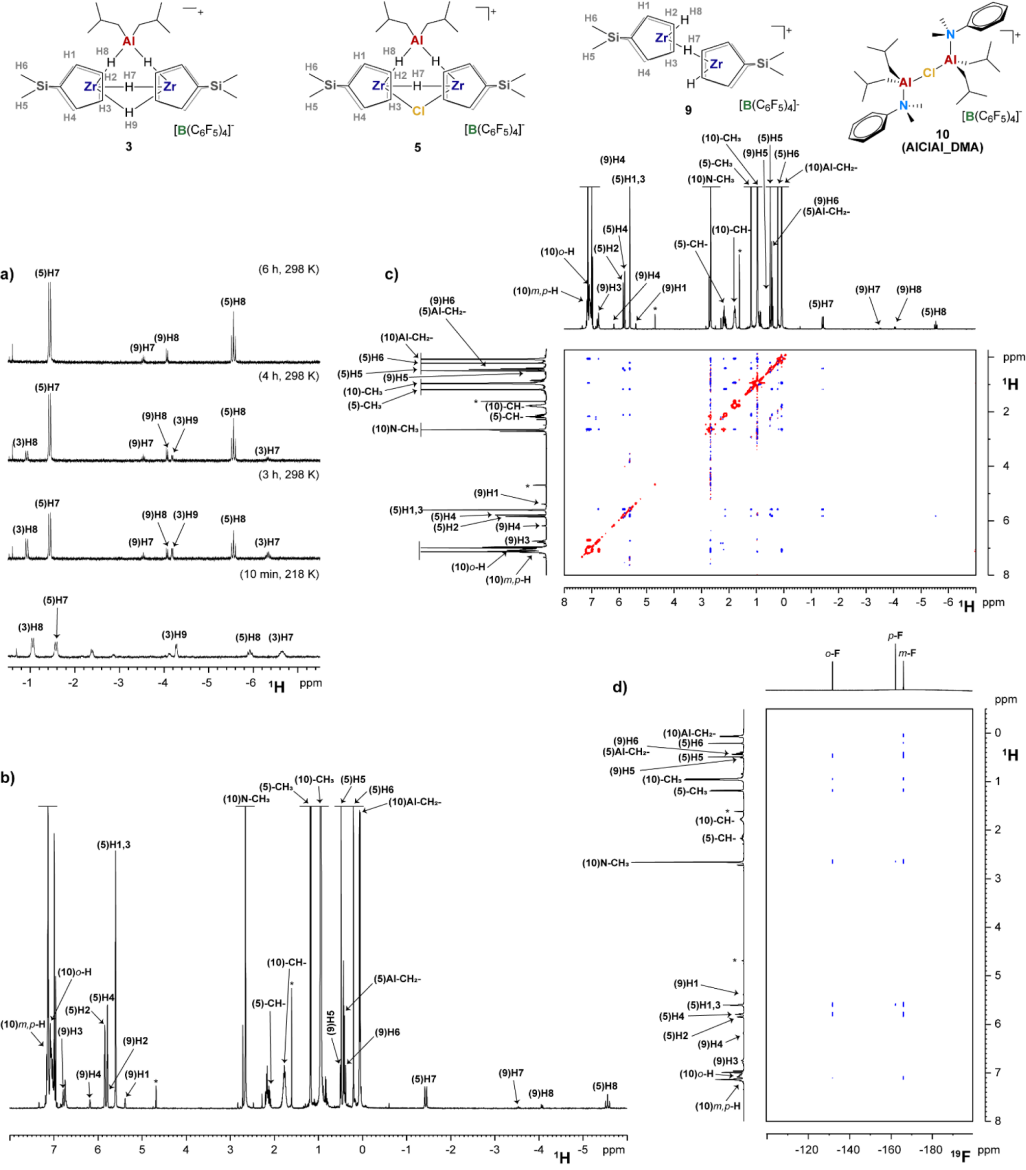
Sections of relevant NMR spectra (chlorobenzene-*d*_5_) of the products obtained from the reaction of (Me_2_SiCp_2_)­ZrCl_2_ with **AlHAl_DMA**: a) comparison of hydride signals in ^1^H NMR spectra at variable temperature and reaction time; b) ^1^H NMR of the final reaction mixture, c) ^1^H NOESY NMR of the final reaction mixture; d) ^19^F–^1^H HOESY NMR of the final reaction mixture. * = *iso*-butene.

The reaction mixture was then allowed to gently warm to 298 K and monitored by ^1^H NMR (Figure 5a). The composition of the mixture varied until it became stable after approximately 6 h (Figure 5a,b). The trinuclear species **3** was completely consumed, whereas **5** remained as the main product, along with a newly formed species. Based on 2D NMR characterization (Figures 5c and S12–S14), the latter species was identified as a dinuclear ZrH–(μ-H)–ZrH cation (**9**), that is, the hydride homologue of **7**.

Layering pentane on a solution of products in chlorobenzene-*d*_5_ allowed the isolation of single crystals of **5** suitable for an X-ray study. The solid-state structure of **5** is shown in Figure 4b. To the best of our knowledge, this is the first example of a solid-state trimetallic structure comprising two group 4 metals and one alkylaluminum moiety. The complex is characterized by the presence of a central planar core comprising the three metal atoms, the chloride, and the three hydrides. The latter were assigned to the residual Q-peaks. While two hydrides are bridging the aluminum and one zirconium (H2 and H3, Figure 4), the third one (H1) is bound to all three metallic atoms. On the contrary, the chloride bridges the two zirconium atoms. Therefore, all metals are pentacoordinated. These coordination environments cause several interesting changes in the bond lengths and angles. As a matter of fact, the alkylaluminum moiety features a smaller C5–Al1–C1 angle (121.0(3)°) with respect to the ones of the monocationic [Al­(DMA)_2_(*i*Bu)_2_]­[B­(C_6_F_5_)_4_] (C5–Al1–C1 = 124.39(8)°) and of **Al_tri_DEA** (C5–Al1–C1 = 131.21(8)°) even though the Al–C bond lengths remain similar (Al1–C1 = 1.972(8) Å, Al1–C5 = 1.976(8) Å for **5**, Al1–C1 = 1.976(2) Å, Al1–C5 = 1.973(2) Å for [Al­(DMA)_2_(*i*Bu)_2_]­[B­(C_6_F_5_)_4_]). On the other hand, the Cp_centroid_-Zr-Cp_centroid_ angles in **5** are similar to those of **7** and **8** (Cp_centroid_-Zr-Cp_centroid_ = 126.8° and 126.8° for **5**, Cp_centroid_-Zr-Cp_centroid_ = 125.4° and 126.1° for **7**, Cp_centroid_-Zr-Cp_centroid_ = 127.1° for **8**), likely due to the rigid structure of the Me_2_SiCp_2_ ligand, while the Zr-Cp_centroid_ bond lengths are significantly longer with respect to **8** (Zr-Cp_centroid_ = 2.184, 2.195, 2.188, and 2.179 Å for **5**, Zr-Cp_centroid_ = 2.164 Å, and 2.162 Å for **8**). Finally, the Zr–Cl bonds are elongated (Zr–Cl = 2.597(2) and 2.596(2) Å for **5**, Zr–Cl = 2.5803(8) and 2.5684(9) Å for **7**, Zr–Cl = 2.5646(5) and 2.5642(7) Å for **8**), likely due to a minor electron deficiency of the complex. Interestingly, the entire crystal structure is stabilized by multiple π–π interactions between the chlorobenzene and two Cp moieties on the Zr1 atom belonging to two different asymmetric units (centroid distances: 3.566 and 3.642 Å).^74^

Several products containing **DMA** fragments were observed, as well. The main N–Me signals exhibit dipolar correlations with an Al-*i*Bu fragment and no Zr-Cp, as observed by ^1^H NOESY NMR (Figure 5c). Dipolar interactions between the protons of this species and the C–F bonds of the [B­(C_6_F_5_)_4_]^−^ anion were detected by ^19^F–^1^H HOESY NMR (Figure 5d), indicating that this species is ionic like Zr/Al adducts. Comparison of NMR chemical shifts with the independently synthesized molecule,^31^ allowed identification of this species as **AlClAl_DMA** (**10**), i.e., the chloride analog of **AlHAl_DMA**. This species likely results from the association of the Al*i*Bu_2_(DMA)Cl byproduct of chloride abstraction with some unreacted [Al*i*Bu_2_(DMA)]^+^.

All of these observations are consistent with the hypothesized activation mechanism involving chloride abstraction by [Al*i*Bu_2_(DMA)]^+^ and Cl/H exchange by the remaining Al*i*Bu_2_(DMA)H subunit of **AlHAl_DMA** (Scheme 2a–e). The central role of the hydride is also consistent with previous reports demonstrating that the chloride-bridged analog **AlClAl_DMA** is unable to fully activate dichloride precatalysts.^31^

### Impurity Scavenging by **AlHAl_DMA**

Finally, the impurity scavenging by **AlHAl_DMA** was explored by deliberately exposing a solution of this cocatalyst in benzene-*d*_6_ to atmospheric oxygen and moisture in a slightly unscrewed J. Young NMR tube. Monitoring the reaction over several days revealed the progressive consumption of **AlHAl_DMA** (Figure 6a). Initially, the Al–H bond disappears, while Al–*i*Bu and Al–DMA groups convert into those of another ionic product. Based on 2D NMR characterization (Figures 6b,c , and S16) and the comparison of chemical shifts with the independently synthesized compound, the latter species was identified as **Al_DMA**, i.e., the mononuclear intermediate toward the synthesis of **AlHAl_DMA**.^31,35,36^

**Figure 6:**
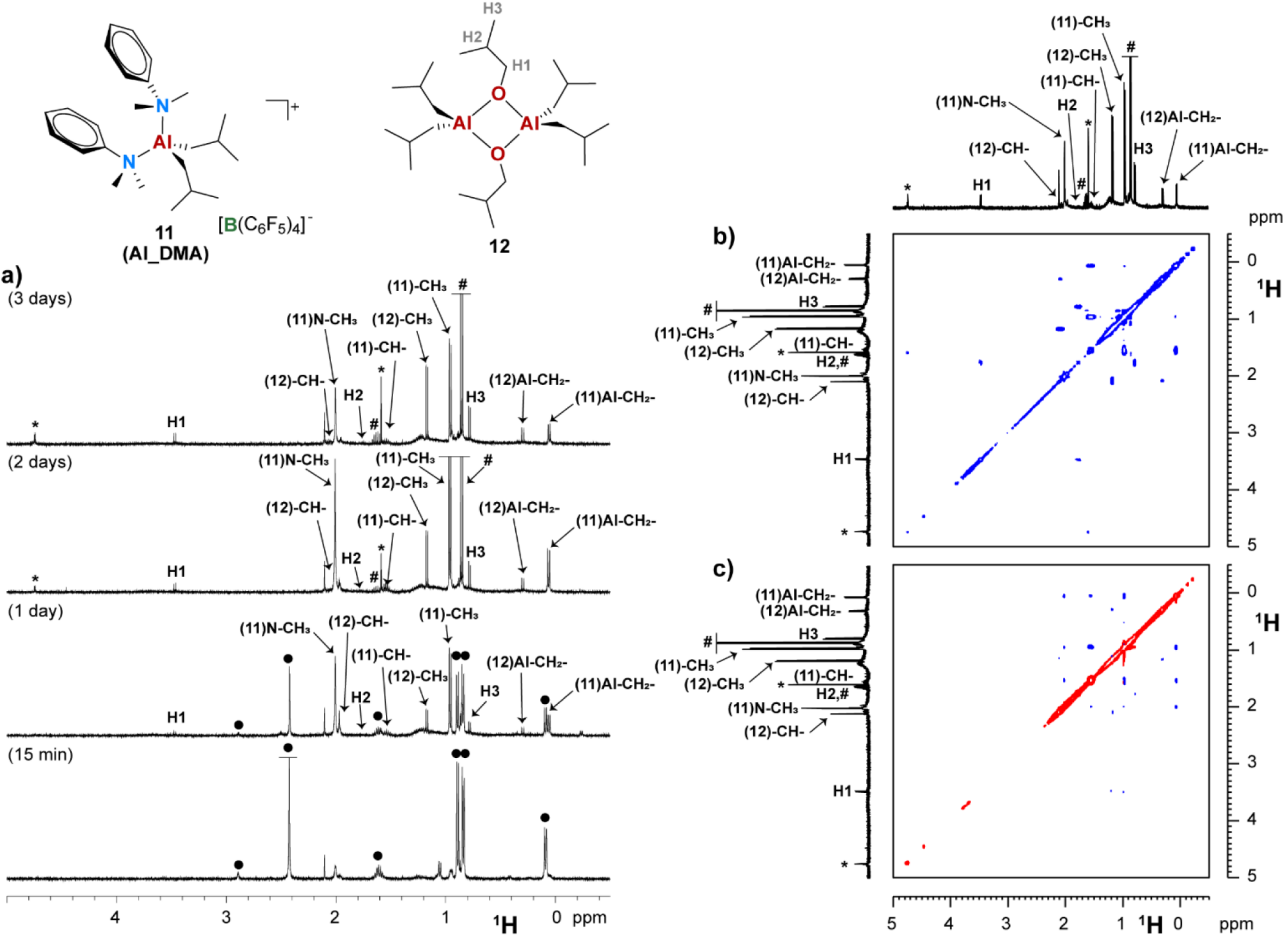
Sections of relevant NMR spectra (chlorobenzene-*d*_5_, 298 K) of the products obtained from exposure of **AlHAl_DMA** to air: a) comparison of hydride signals in ^1^H NMR spectra at variable reaction time; b) ^1^H COSY NMR; and c) ^1^H NOESY NMR. • = **AlHAl_DMA**; ^#^ = *iso*-butane; * = *iso*-butene.

Along with **Al_DMA**, other species formed, such as *iso*-butane (deriving from hydrolysis of Al–*i*Bu bonds) and *iso*-butene via β-H elimination from Al−*i*Bu groups. An Al–O*i*Bu species likely deriving from partial oxidation of Al-alkyls, also formed as suggested by the diagnostic peaks at δ­(^1^H) = 3.47 ppm and δ­(^13^C) = 70.8 ppm:^75,76^ this was identified as the neutral dinuclear adduct **12** by 2D NMR characterization (Figures 6a,b and S15) and comparison with the independently synthesized species by reaction of TIBA and *iso*-butanol (Figure S17, Scheme 6).

**Scheme 6:**
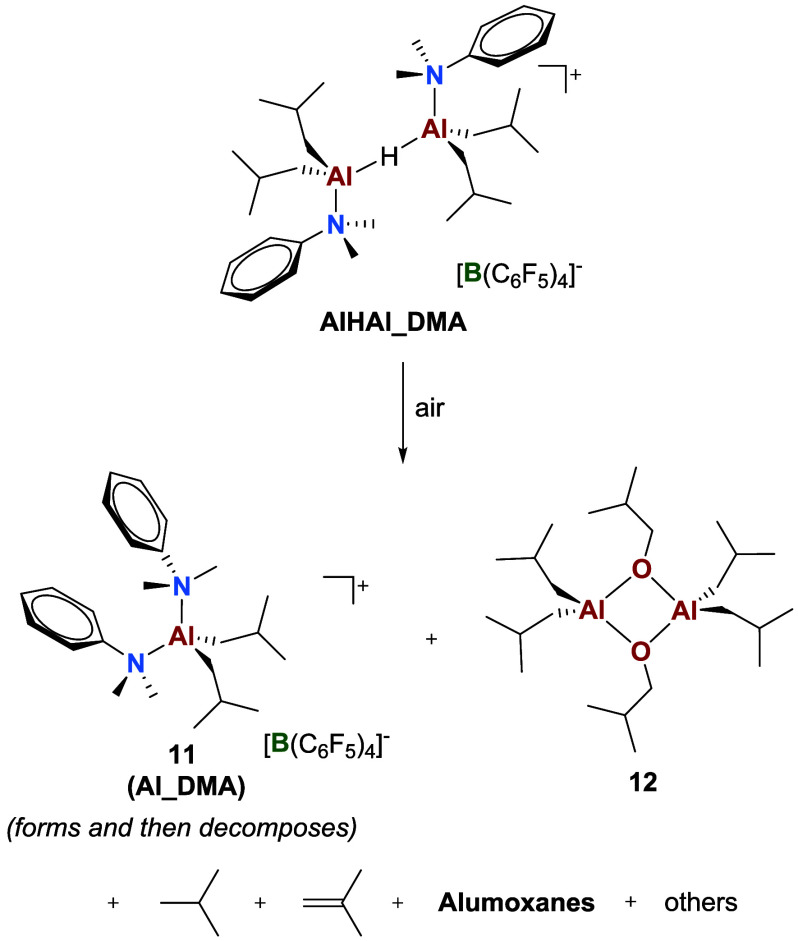
Reactivity of **AlHAl_DMA** upon Exposure to Air

Over time, these products became more evident in the NMR spectra, as their concentration also increased, while **Al_DMA** decomposition occurred (Figure 6a). Eventually, broad resonances also became evident around 0.5–1.4, 2, and 7.0–7.4 ppm likely corresponding to *iso*-butylalumoxane oligomers decorated by **DMA** ligands (Figure 6a). The presence of these broad signals can be better evidenced by ^1^H PGSE experiments: since the signal attenuation of smaller neutral molecules is more marked at increasing gradient strength (G),^41,42^ it is possible to obtain an NMR spectrum evidencing the resonances of the large alumoxane oligomers along with some remaining ionic aggregates of **Al_DMA** (Figure S16).

In previous reports, we showed that appreciable catalytic activity could be obtained using **AlHAl_DMA** at very low catalyst (and scavenger) concentrations, while TIBAL/organic borate salts were ineffective under these conditions.^31,35^ We attributed this observation to the enhanced scavenging ability of this activator. The results reported here suggest that the excellent impurity-scavenging properties of **AlHAl_DMA** are primarily due to the highly reactive Al-hydride moiety that reacts with catalyst poisons like water and oxygen more rapidly than Al–*i*Bu groups, which are also present in TIBAL.

## Conclusions

The activating and impurity scavenging capabilities of **AlHAl_DMA**, an Al-alkyl borate salt exhibiting similar performance to MAO in polymerization as a stand-alone cocatalyst, were investigated by exploiting the well-defined structure of this novel cocatalyst as well as three stable AAB salts with tricoordinate Al-alkyl cations as models for the reactive [Al*i*Bu_2_(DMA)]^+^ subunit. The reaction of the representative (Me_2_SiCp_2_)­ZrCl_2_ precatalyst with the model tricoordinate AAB salts allowed the isolation of singly bridged Zr–(μ-Cl)–Al heterodinuclear adducts, which represent plausible intermediates of Cl-abstraction from zirconium. The addition of DIBAL-H to these species led to the formation of several multinuclear adducts with bridging hydride and chloride groups between Zr and Al.

Analogous products were also observed upon reacting (Me_2_SiCp_2_)­ZrCl_2_ with **AlHAl_DMA**, demonstrating that they are relevant intermediates of precatalyst activation with this novel cocatalyst. The lack of any detected Zr–*i*Bu fragment suggests that **AlHAl_DMA** works as a hydride source, leading to Zr–H active species (Schemes 2a–e and 7). The hydride moiety of this cocatalyst appears to be relevant also with respect to its scavenging ability, as it is particularly reactive toward catalyst poisons like oxygen and water.

**Scheme 7:**
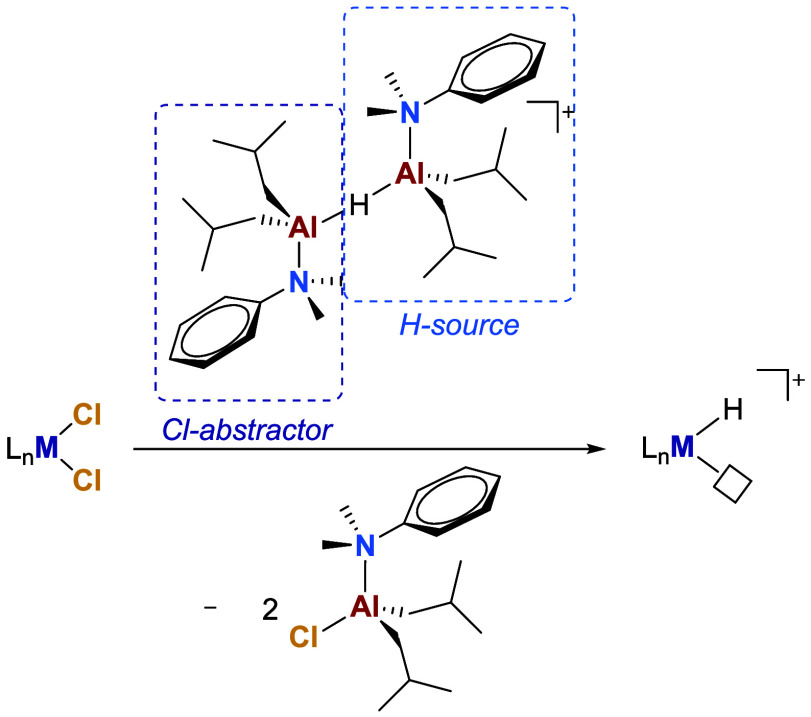
Roles of **AlHAl_DMA** Subunits in Precatalyst Activation

The results reported herein therefore provide a detailed understanding of the reactivity of **AlHAl_DMA** as a cocatalyst in olefin polymerization, which could also be exploited in future studies aimed at further refining the properties of this system. Furthermore, the isolated species, including those derived from reaction with tricoordinate AAB salts, are relevant in the broader context of mechanistic studies of olefin polymerization due to the relevance of M/Al heteronuclear adducts. Importantly, similar species are also relevant intermediates for other applications of group 4 metal and Al organometallics like zirconium-catalyzed carboaluminations,^77^ hydrodefluorination,^73^ and more.^78^

## Experimental Part

### Materials and Methods

All manipulations were performed under rigorous exclusion of oxygen and moisture in flame-dried Schlenk-type glassware interfaced to a high-vacuum line (<10^–5^ Torr), or in a nitrogen-filled MBraun glovebox (<0.5 ppm of O_2_). Molecular sieves (4 Å, MS) were activated for 24 h at ca. 200–230 °C under a dynamic vacuum. Benzene-*d*_6_ and toluene-*d*_8_ were freeze-pump-thaw degassed on a high-vacuum line, dried over Na/K alloy, vacuum-transferred to a dry storage tube with a PTFE valve, and stored over activated MS in the glovebox. Chlorobenzene-*d*_5_ was freeze-pump-thaw degassed on the high-vacuum line, dried over CaH_2_, vacuum-transferred to a dry storage tube with a PTFE valve, and stored over activated MS, protected from light, in the glovebox. TTB and AB were obtained from Boulder Scientific Co. and used as received. (Me_2_SiCp_2_)­ZrCl_2_ DIBAL-H, and TIBAL were purchased from Merck. **AlHAl_DMA, Al_tri_HMDSO**, **Al_tri_DEA**, and **Al_tri_NHC** were synthesized according to previously reported procedures.^31,32,35^

NMR experiments were performed using a Bruker Avance III HD 400 instrument equipped with a smart probe (400 MHz for ^1^H). ^1^H NMR spectra were referenced to the residual protons of the deuterated solvent used; ^13^C NMR spectra were referenced internally to the D-coupled ^13^C resonances of the NMR solvent. To describe the multiplicity of the signals, the following abbreviations are used: s, singlet; bs, broad singlet; d, doublet; bd, broad doublet; dd, doublet of doublets; t, triplet; and m, multiplet.

Diffusion NMR measurements were performed at 298 K in toluene-*d*_8_ using the standard double-stimulated echo pulse sequence without spinning. The shape of the gradients was rectangular, their duration (d) was 4 ms, and their strength (G) was varied during the experiments. All the spectra were acquired by using 16K data points, between 16 and 128 scans, a spectral width of 6000 Hz, an acquisition time of 1.3 s, and a relaxation delay of 2 s per transient. Toluene was used as an internal standard to account for temperature and viscosity fluctuations; its *D*_t_ was calibrated by using an external sample of HDO in D_2_O under the same exact conditions.^79^ The *D*_t_ data were treated as described in the literature to derive the hydrodynamic dimensions.^42^

X-ray diffraction patterns of crystals were recorded using a Bruker D8 Venture diffractometer equipped with an Incoatec ImuS3.0 microfocus sealed-tube MoKα (λ = 0.71073 Å) source and a CCD Photon II detector. The analyses were carried out at low temperature (120–150 K range) using an Oxford Cryosystems Cryostream 800 cooler. The data collected through generic φ and ω scans were integrated and reduced using Bruker AXS V8 Saint software. The structures were solved and all the thermal parameters were anisotropically refined using the SHELXT and SHELXL packages of the Bruker APEX3 software.^80,81^ To refine chlorobenzene molecules in the asymmetric unit of the crystals, the disorder model ICAHEB present in the DSR plugin of the Bruker APEX3 software was employed.^82^

## Synthetic Details

### Chloride Abstraction

**1 (HMDSO)**. *Experiment 1, low T*. Cold chlorobenzene-d_5_ (600 μL; 245 K) was added to a solid mixture of Me_2_SiCp_2_ZrCl_2_ (10 μmol) and **Al_tri_HMDSO** (30 μmol). The resulting solution was rapidly cooled to ≈200 K using an acetone/liquid nitrogen bath and then transferred to the precooled NMR probe at 223 K. The addition resulted in an immediate color change from yellow to almost colorless, indicating that a rapid reaction occurred.

*Experiment 2, RT.* Cold chlorobenzene-*d*_5_ (600 μL; 245 K) was added to a solid mixture of Me_2_SiCp_2_ZrCl_2_ (12.9 μmol) and **Al_tri_HMDSO** (12.6 μmol). The resulting solution was rapidly cooled to ≈200 K using an acetone/liquid nitrogen bath and then transferred to the NMR probe at 298 K. The addition resulted in an immediate color change from yellow to almost colorless, indicating that a rapid reaction occurred.

**1.** ^1^H NMR (400 MHz, 223 K, chlorobenzene-*d*_5_): 6.85 (t, 4H, aromatic H2,3), 5.43 (t, 4H, aromatic H1,4), 2.09 (m, 2H, CH), 1.20 (d, 12H, CH_3_), 0.68 (s, 6H, Si–CH_3_), 0.57 (d, 4H, Al–CH_2_) ppm. ^13^C NMR (100 MHz, 223 K, chlorobenzene-*d*_5_): 132.5 (aromatic C2,3), 121.7 (aromatic C1,4), 114.3 (aromatic C–Si), 27.7 (CH_3_), 25.9 (CH), 24.7 (Al–CH_2_), −6.5 (Si–CH_3_) ppm.

**HMDSO.** ^1^H NMR (400 MHz, 223 K, chlorobenzene-*d*_5_): 0.15 (s, 18H, Si–CH_3_) ppm. ^13^C NMR (100 MHz, 223 K, chlorobenzene-*d*_5_): 2.2 (Si–CH_3_) ppm.

**2_DEA and 2_NHC.** Cold chlorobenzene-*d*_5_ (600 μL; 245 K) was added to a solid mixture of Me_2_SiCp_2_ZrCl_2_ (12.9 μmol) and **Al_tri_DEA/NHC** (12.6 μmol). The resulting solution was rapidly cooled to ≈200 K using an acetone/liquid nitrogen bath and then transferred to the precooled NMR probe at 223 K. The addition resulted in an immediate color change from yellow to almost colorless, indicating that a rapid reaction occurred.

**2_DEA.** ^1^H NMR (400 MHz, 253 K, chlorobenzene-*d*_5_): 7.35 (bd, 2H, aromatic *m*-CH), 7.25 (bd, 1H, aromatic *p*-CH), 7.14 (bs, 2H, aromatic *o*-CH), 6.97 (bs, 4H, aromatic H2,3), 5.66 (s, 4H, aromatic H1,4), 3.44 (m, 2H, N–CH_2_), 3.27 (m, 2H, N–CH_2_), 1.87 (m, 2H, CH), 1.13 (bs, 12H, CH_3_), 1.03 (bs, 6H, N–CH_2_C*H*_3_), 0.57 (bs, 6H, Si–CH_3_), 0.30 (bd, 4H, AlCH_2_) ppm. ^13^C NMR (100 MHz, 253 K, chlorobenzene-*d*_5_): 133.8 (aromatic N–C), 130.0 (aromatic *m*-C), 129.9 (aromatic C2,3), 129.8 (aromatic *p*-C), 116.8 (aromatic C1,4), 110.0 (aromatic C–Si), 48.4 (N–CH_2_), 27.9 (CH_3_), 26.0 (CH), 23.6 (AlCH_2_), 9.01 (N–CH_2_ *C*H_3_), −6.26 (Si–CH_3_) ppm.

**2_NHC.** ^1^H NMR (400 MHz, 223 K, chlorobenzene-*d*_5_): 7.55 (t, 2H, aromatic *p*-H), 7.28 (m, 4H, aromatic *m*-H), 7.19 (bs, 2H, NC*H*C*H*N), 6.93 (bs, 4H, aromatic H3), 6.55 (bs, 2H, aromatic H2), 5.54 (bs, 4H, aromatic H1,4), 2.58 (m, 4H, H7,9), 1.81 (m, 2H, CH), 1.46 (bd, 12H, H8), 1.16 (bd, 12H, CH_3_), 1.13 (bd, 6H, H10), 0.92 (bd, 6H, CH_3_), 0.70 (bs, 3H, H5), 0.52 (bs, 3H, H6), −0.01 (m, 4H, Al–CH_2_) ppm. ^13^C NMR (100 MHz, 223 K, chlorobenzene-*d*_5_): 166.8 (N*C*N), 145.7 (aromatic *o*-C), 133.3 (aromatic *C*–N), 131.9 (aromatic *p*-C), 130.9 (aromatic C3), 128.2 (aromatic C2), 126.2 (N*C*H*C*HN), 124.7 (aromatic *m*-C), 117.3 (aromatic C1,4), 114.9 (aromatic C1,4), 110.5 (aromatic C–Si), 29.1 (C7,9), 28.6 (CH_3_), 27.4 (CH_3_), 25.9 (C8,10), 25.6 (CH), 23.5 (Al-CH_2_), 22.6 (C8,10), −6.1 (C5, C6), −6.3 (C5, C6) ppm.

### Reaction with DIBAL-H

An excess of DIBAL-H (≈2.5 equiv) was added to a preformed solution of **2_DEA** and **2_NHC** (11.2 μmol) in chlorobenzene-*d*_5_ (600 μL). The resulting solution was rapidly cooled to ≈200 K using an acetone/liquid nitrogen bath and then transferred to the precooled NMR probe at 223 K. The addition resulted in an immediate color change from yellow to colorless, indicating that a rapid reaction occurred.

### Complex **3**

^1^H NMR (400 MHz, 223 K, chlorobenzene-*d*_5_): 6.47 (m, 4H, H3), 6.12 (m, 4H, H2), 5.70 (m, 4H, H1), 5.46 (m, 4H, H4), 1.95 (m, 2H, CH), 1.08 (m, 12H, CH_3_), 0.39 (s, 6H, H5), 0.29 (m, 4H, Al–CH_2_), 0.17 (s, 6H, H6), −0.92 (d, 2H, H8), −4.18 (d, 1H, H9), −6.32 (m, 1H, H7) ppm. ^13^C NMR (100 MHz, 223 K, chlorobenzene-*d*_5_): 118.5 (C3), 110.7 (C1), 110.6 (C2), 107.3 (C4), 27.9 (CH_3_), 26.4 (Al–CH_2_), 25.7 (CH), −6.1 (C5), −7.5 (C6) ppm.

### Complex **4**

^1^H NMR (400 MHz, 223 K, chlorobenzene-*d*_5_): 6.40 (m, 4H, H2,3), 5.56 (m, 4H, H1,4), 0.38 (s, 6H, H5,6), 0.08 (t, 1H, H7), −2.14 (d, 2H, H8) ppm.

### Complex **5**

^1^H NMR (400 MHz, 223 K, chlorobenzene-*d*_5_): 5.84 (m, 4H, H2), 5.79 (m, 4H, H4), 5.60 (m, 4H, H3), 5.60 (m, 4H, H1), 2.12 (m, 2H, CH), 1.17 (d, 12H, CH_3_), 0.48 (s, 6H, H5), 0.42 (m, 4H, Al–CH_2_), 0.21 (s, 6H, H6), −1.44 (d, 1H, H7), −5.54 (t, 2H, H8) ppm. ^13^C NMR (100 MHz, 223 K, chlorobenzene-*d*_5_): 126.5 (C3), 109.9 (C2), 107.8 (C1), 105.9 (C4), 27.7 (CH_3_), 27.1 (CH), 25.1 (Al–CH_2_), −5.4 (C5), −7.5 (C6) ppm.

### Complex **6**

^1^H NMR (400 MHz, 223 K, chlorobenzene-*d*_5_): 6.14 (m, 8H, H2,3), 5.47 (m, 8H, H1,4), 2.05 (m, 4H, CH), 1.12 (d, 24H, CH_3_), 0.39 (s, 12H, H5,6), 0.36 (m, 8H, Al-CH_2_), −3.05 (d, 4H, H8), −4.48 (m, 1H, H7) ppm. ^13^C NMR (100 MHz, 223 K, chlorobenzene-*d*_5_): 114.5 (C2,3), 105.9 (C1,4), 27.9 (CH_3_), 26.8 (CH), 23.9 (Al–CH_2_), −6.1 (C5,6) ppm.

### Reaction with **AlHAl_DMA**

A solution of **AlHAl_DMA** (12.9 μmol) in 200 μL of chlorobenzene-*d*_5_ was added to a solution of Me_2_SiCp_2_ZrCl_2_ (12.9 μmol) in 400 μL of chlorobenzene-*d*_5_. The resulting solution was rapidly cooled to ≈200 K using an acetone/liquid nitrogen bath and then transferred to the precooled NMR probe at 223 K. The addition resulted in an immediate color change from colorless to pale yellow, indicating that a rapid reaction occurred.

### Complex **9**

^1^H NMR (400 MHz, 223 K, chlorobenzene-*d*_5_): 6.79 (m, 4H, H3), 6.17 (m, 4H, H4), 5.78 (m, 4H, H2), 5.38 (m, 4H, H1), 0.51 (s, 6H, H6), 0.38 (s, 6H, H5), −3.50 (t, 1H, H7), −4.07 (d, 2H, H8) ppm. ^13^C NMR (100 MHz, 223 K, chlorobenzene-*d*_5_): 122.9 (C3), 119.0 (C3), 109.8 (C1), −6.40 (C5), −6.71 (C6) ppm.

### AlClAl_DMA (**10**)

^1^H NMR (400 MHz, 223 K, chlorobenzene-*d*_5_): 7.14 (m, 6H, aromatic *m*-H, *p*-H), 7.07 (bd, 4H, aromatic *o*-H), 2.66 (s, 12H, N–CH_3_), 1.77 (m, 4H, CH), 0.95 (d, 24H, CH_3_), 0.07 (m, 8H, Al–CH_2_) ppm. ^13^C NMR (100 MHz, 223 K, chlorobenzene-*d*_5_): 129.5 (aromatic *m*-C, *p*-C), 120.5 (aromatic *o*-C), 45.9 (N–CH_3_), 27.7 (CH_3_), 25.9 (CH), 22.4 (Al-CH_2_) ppm.

### Scavenging Ability of **AlHAl_DMA**

A solution of **AlHAl_DMA** (6 μmol) in 600 μL of benzene-*d*_6_ was prepared under the inert atmosphere of a glovebox in a J. Young NMR tube. Subsequently, the tube was brought outside the glovebox and slightly unscrewed to allow slow contamination with air. The mixture was monitored by NMR over several days.

**12.** ^1^H NMR (400 MHz, 298 K, benzene-*d*_6_): 3.47 (d, 4H, H1), 2.06 (m, 4H, −CH−), 1.76 (m, 2H, H2), 1.17 (d, 24H, −CH_3_), 0.78 (d, 12H, H3), 0.30 (d, 8H, Al–CH_2_−) ppm. ^13^C NMR (100 MHz, 298 K, benzene-*d*_6_): 70.8 (H1), 28.4 (−CH_3_), 25.8 (−CH−), 19.0 (H3) ppm.

## Supplementary Material











## Notes

Characterization details (PDF)
NMR spectra (ZIP)
Crystal structures (CIF) (CIF) (CIF)
CCDC 2466601 (**8**), 2466602 (**7**), and 2466603 (**5**) contain the supplementary crystallographic data for this paper
