# Supplementary material for: Mechanism of Action of MAO’s Molecular Cousin
Source: ACS Catal. 2025 Oct 10;15(21):17658–71. doi: 10.1021/acscatal.5c05698 (PMC12606257; doi:10.1021/acscatal.5c05698)
Supplement: Supplementary file 1 [file cs5c05698_si_001.pdf]

Supporting Information

Mechanism of Action of MAO's *Molecular Cousin*

Gaia Urciuoli,[a,b,c] Francesco Zaccaria,[a,c,*] Cristiano Zuccaccia,[b,c,*] Roberta Cipullo,[a,c] Peter H. M. Budzelaar,[a] Gabriel Menendez Rodriguez,[b] Leonardo Tensi,[d] Antonio Vittoria,[a] Christian Ehm,[a,c] Alceo Macchioni,[b,c] Vincenzo Busico[a,c]

[a] Department of Chemical Sciences, Federico II University of Naples, via Cinthia, 80126 Napoli, Italy.
[b] Department of Chemistry, Biology and Biotechnology, University of Perugia and CIRCC, via Elce di Sotto 8, 06123 Perugia, Italy
[c] DPI, P.O. Box 902, 5600 AX Eindhoven, the Netherlands
[d]Department of Pharmaceutical Sciences, University of Perugia, Via del Liceo 1, 06123 Perugia, Italy
E-mail: francesco.zaccaria@unina.it, cristiano.zuccaccia@unipg.it

Table of Content

1. Additional NMR spectra 2
 1.1 Chloride abstraction 2
 1.2 Reaction with DIBAL-H 10
 1.3 Reaction with AlHAl_DMA 13
 3.4 Scavenging ability of AlHAl_DMA 16
2. X-ray crystallographic details 18

1. Additional NMR spectra

1.1 Chloride abstraction

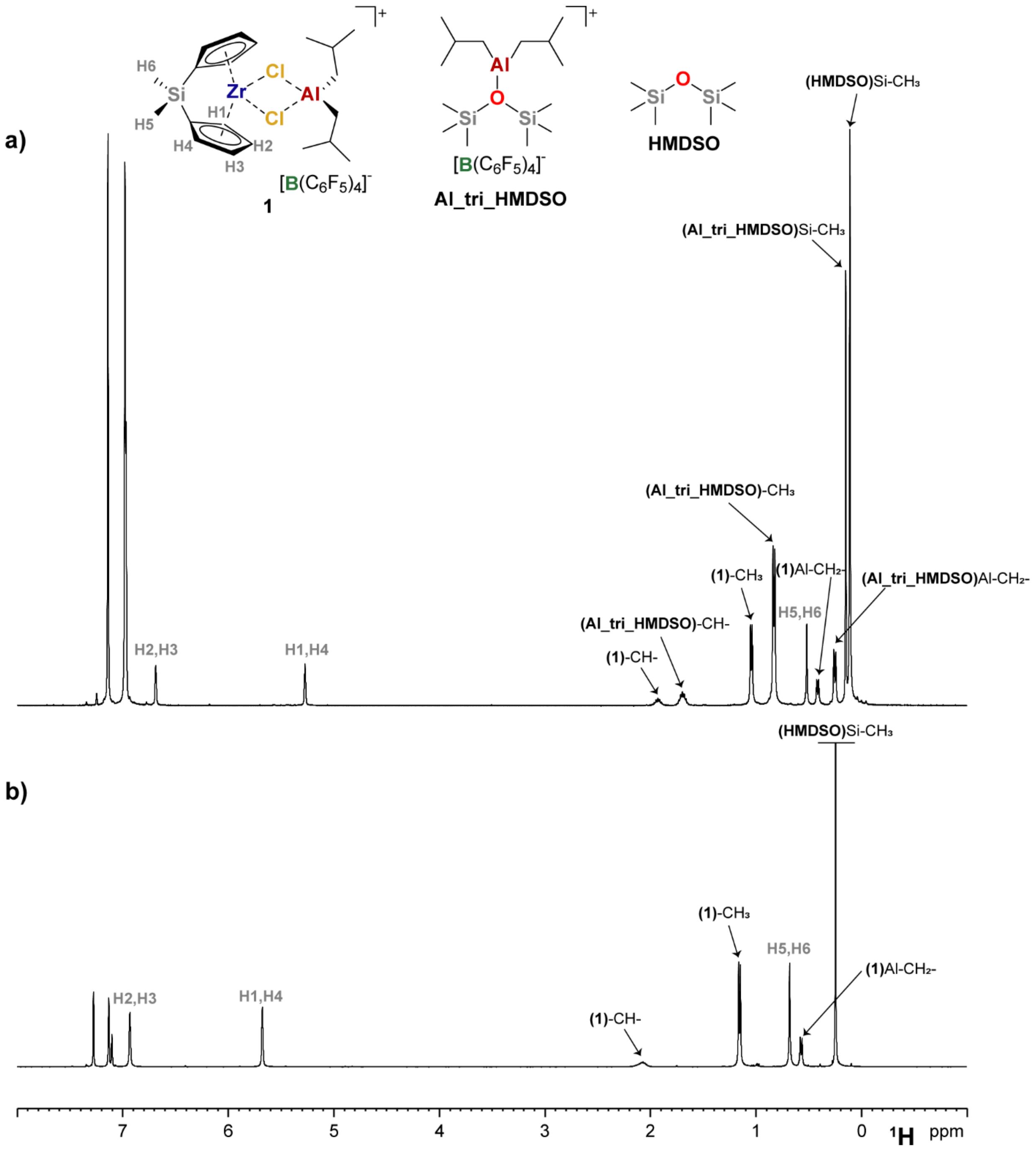

Figure S1. ^{1}H NMR spectra of the products of the reaction between $(Me_2SiCp_2)ZrCl_2$ and **Al_tri_HMDSO** at a) 223 K and b) 298 K in chlorobenzene-d_5.

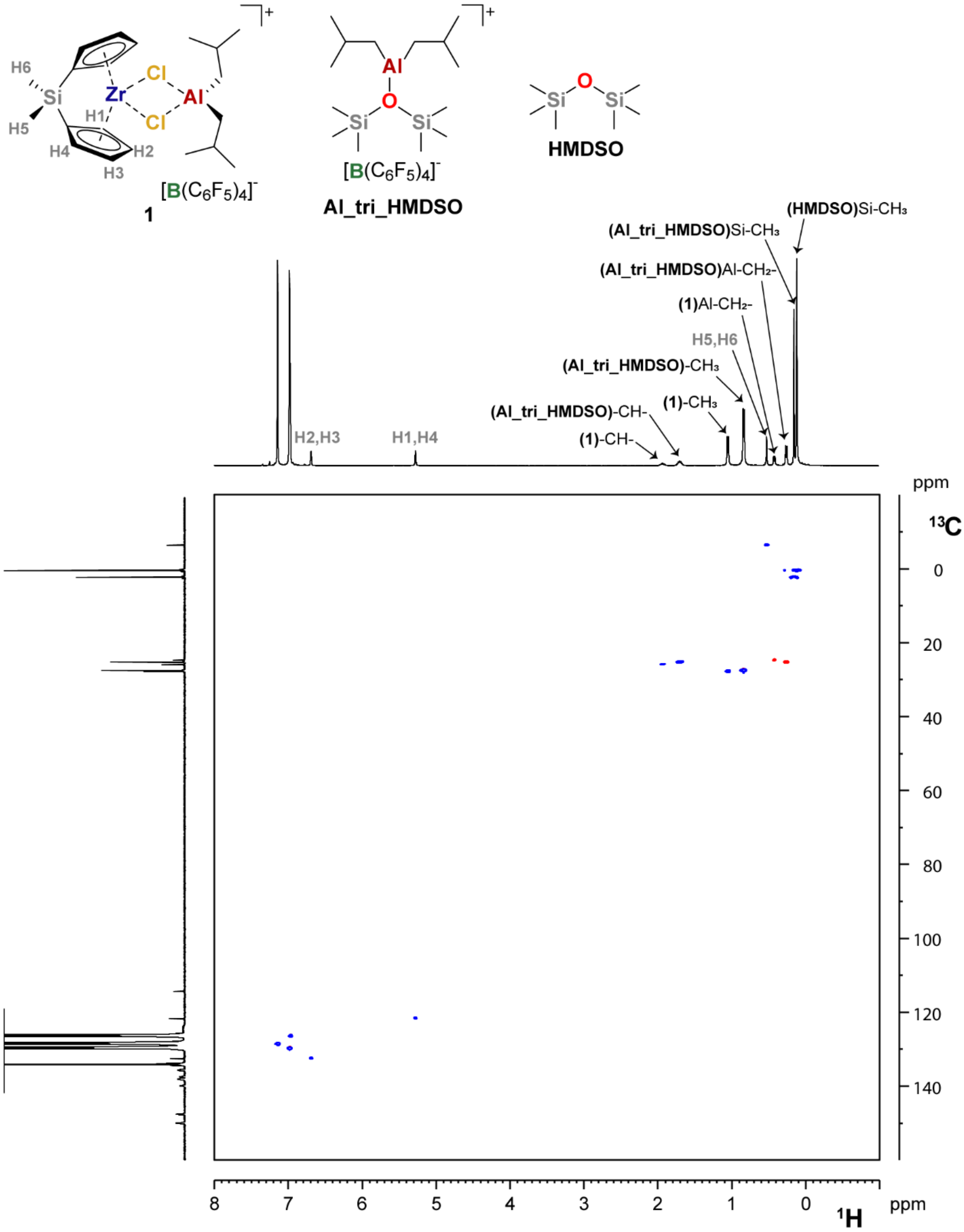

Figure S2. ^{1}H-^{13}C HSQC NMR spectrum of the products of the reaction between $(Me_2SiCp_2)ZrCl_2$ and **Al_tri_HMDSO** at 223 K in chlorobenzene-d_5.

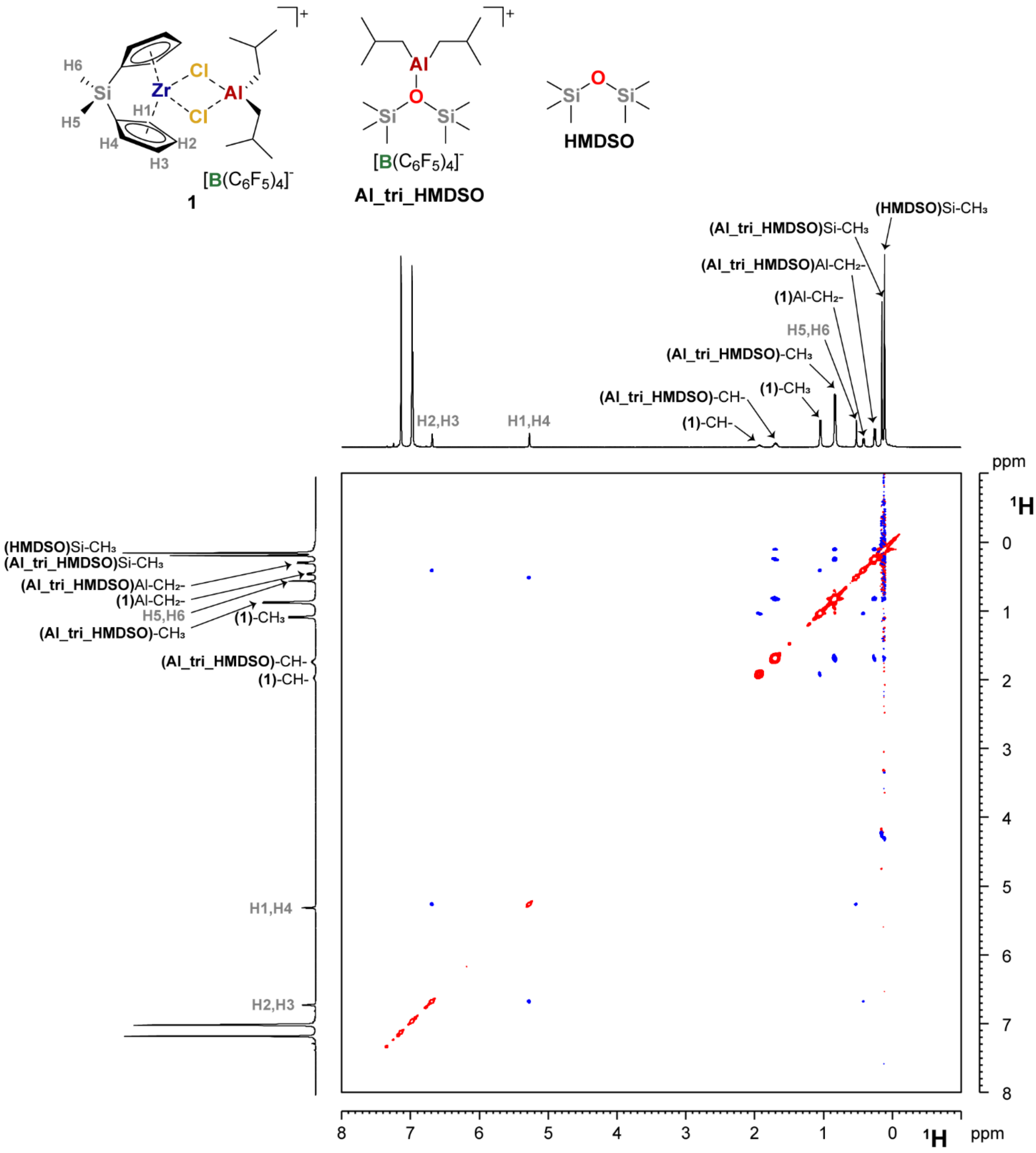

Figure S3. ^{1}H ROESY NMR spectrum of the products of the reaction between $(Me_2SiCp_2)ZrCl_2$ and **Al_tri_HMDSO** at 223 K in chlorobenzene-d_5.

a)

b)

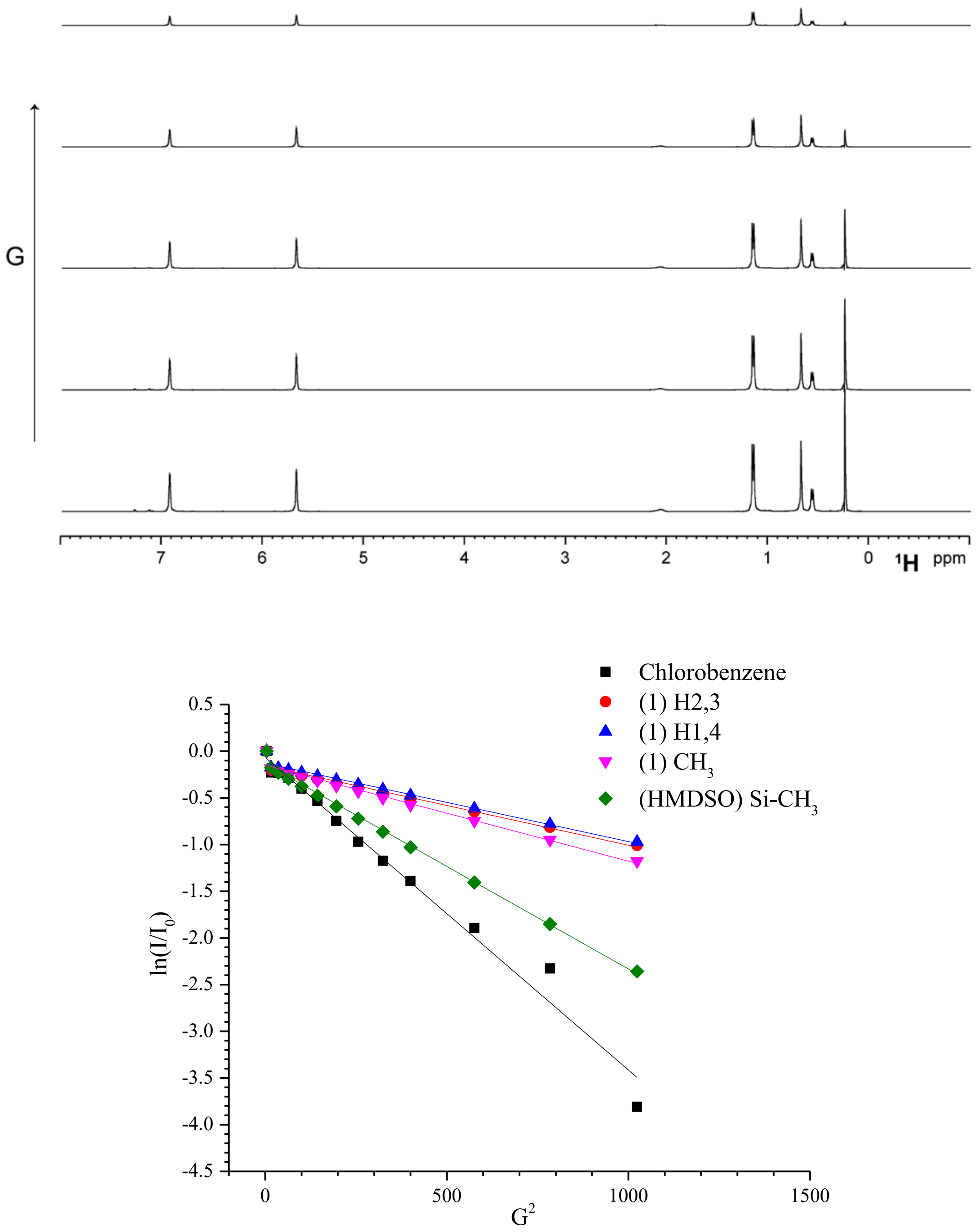

Figure S4. a) ^{1}H PGSE NMR spectra at increasing gradient strength (G) of the products of the reaction between $(Me_2SiCp_2)ZrCl_2$ and **Al_tri_HMDSO** at 298 K in chlorobenzene-d_5 and b) corresponding Ln(I/I_0) vs G^2 plot highlighting that HMDSO diffuses separately from **1**.

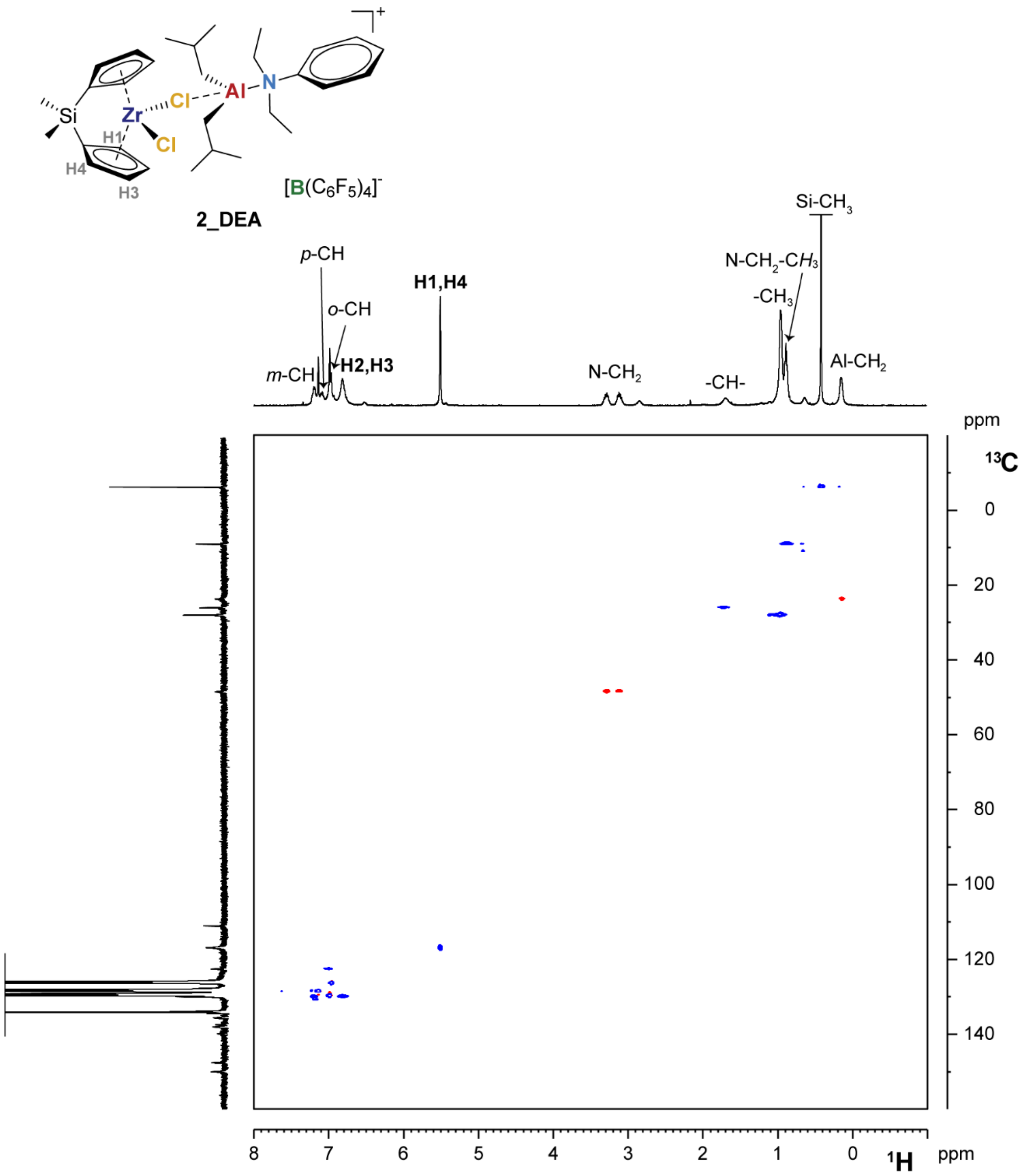

Figure S5. ^{1}H-^{13}C HSQC NMR spectrum of **2_DEA** at 253 K in chlorobenzene-d_5

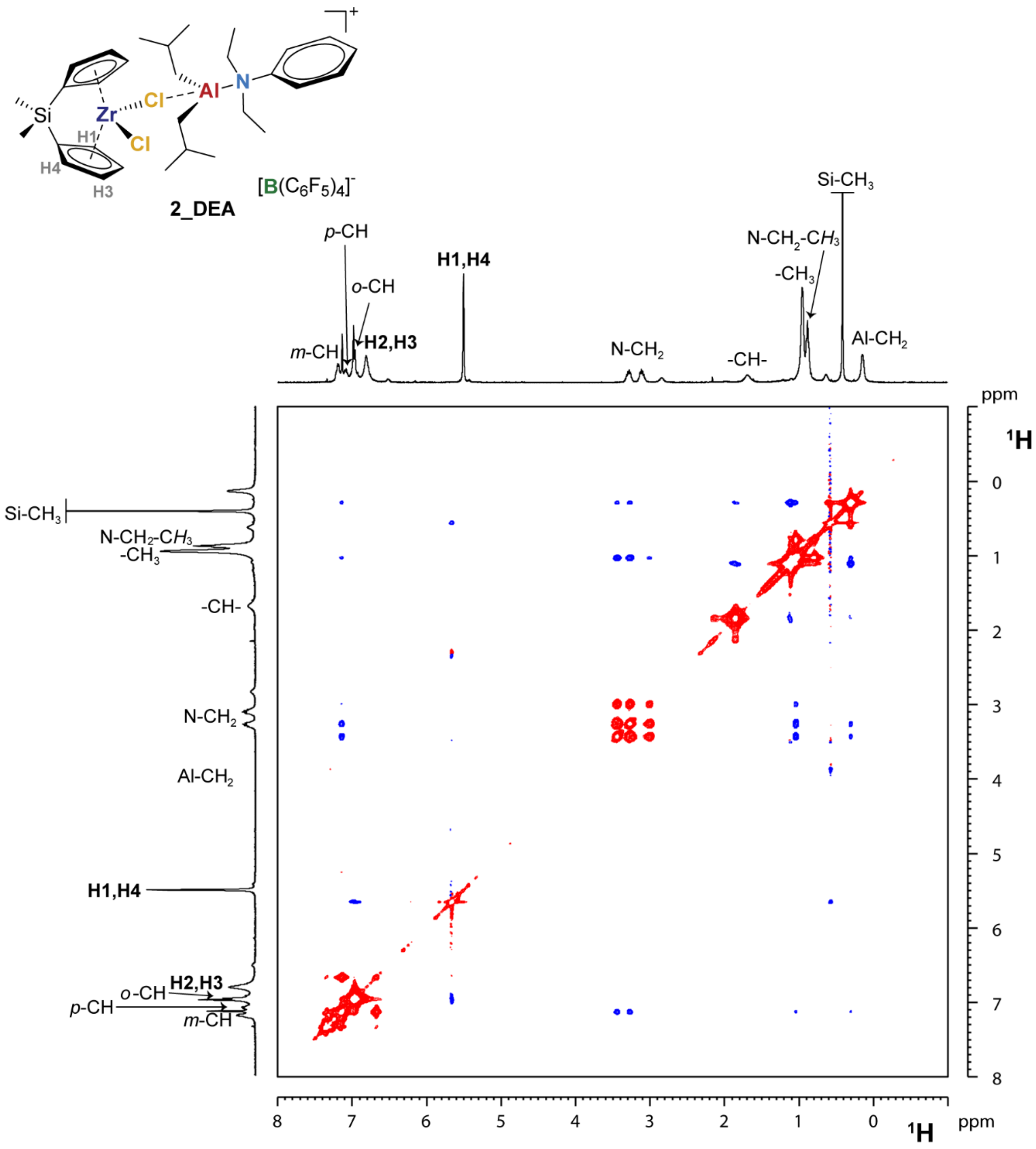

Figure S6. ^{1}H ROESY NMR spectrum of **2_DEA** at 253 K in chlorobenzene-d_5

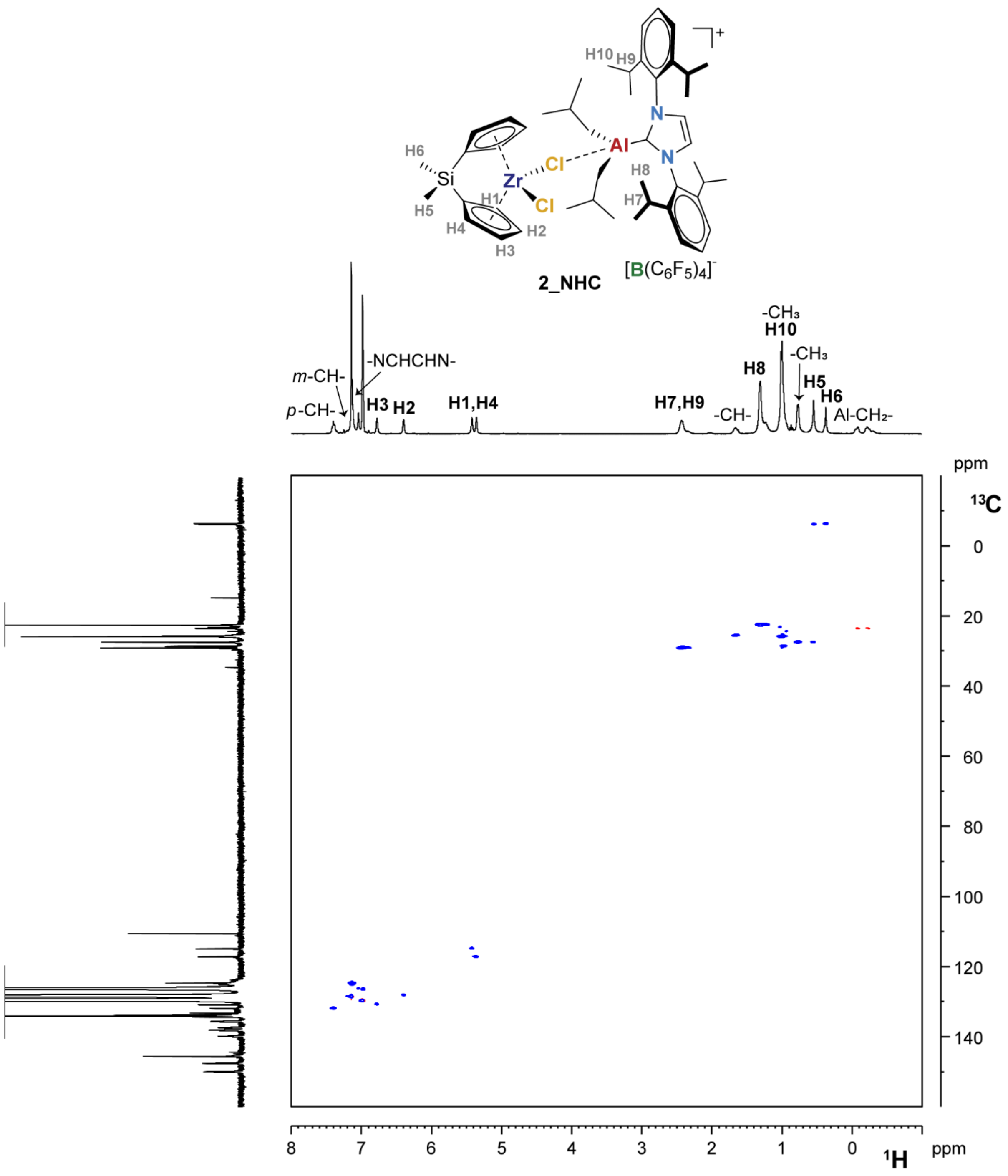

Figure S7. ^{1}H-^{13}C HSQC NMR spectrum of **2_NHC** at 223 K in chlorobenzene-d_5

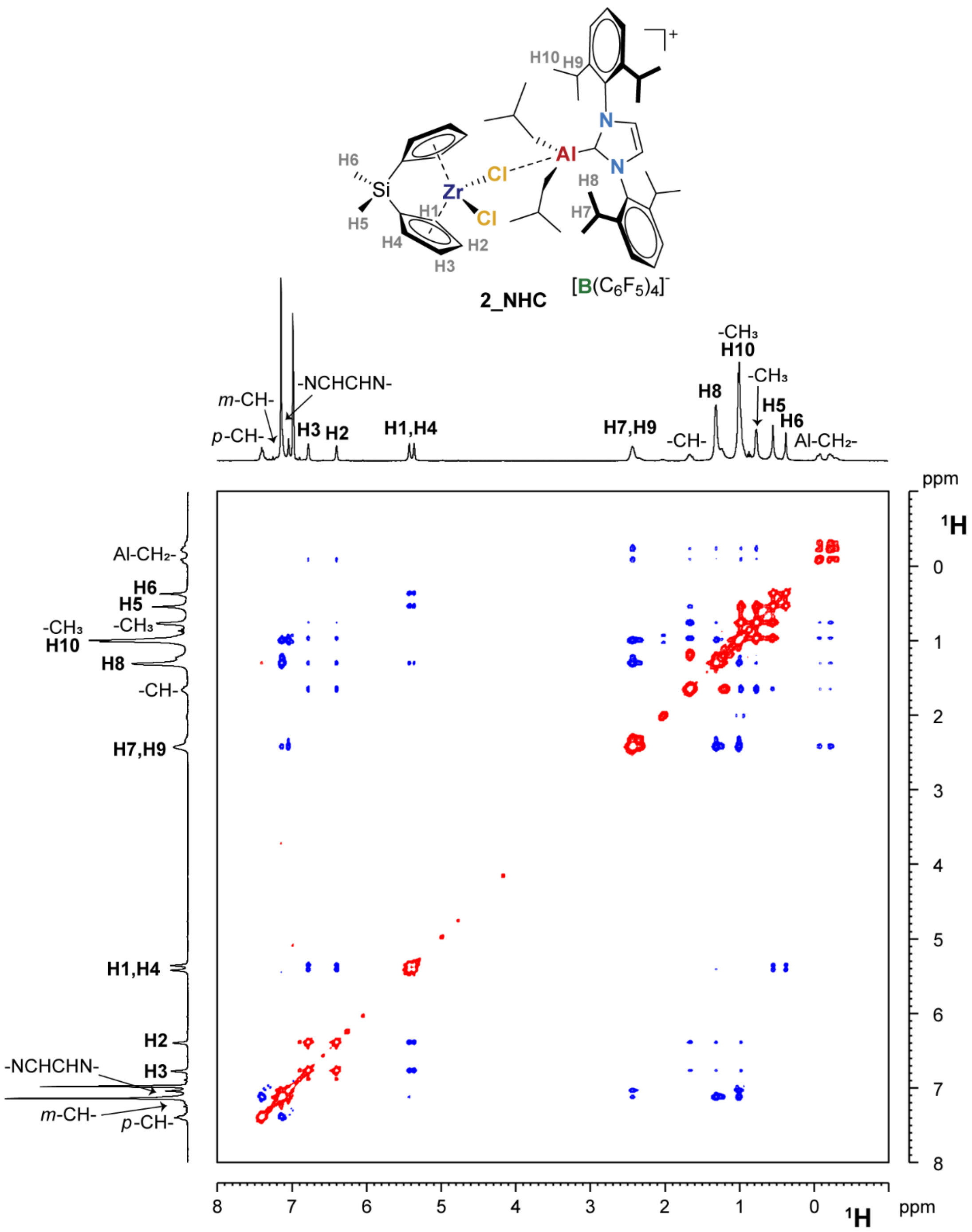

Figure S8. ^{1}H ROESY NMR spectrum of **2_NHC** at 223 K in chlorobenzene-d_5

1.2 Reaction with DIBAL-H

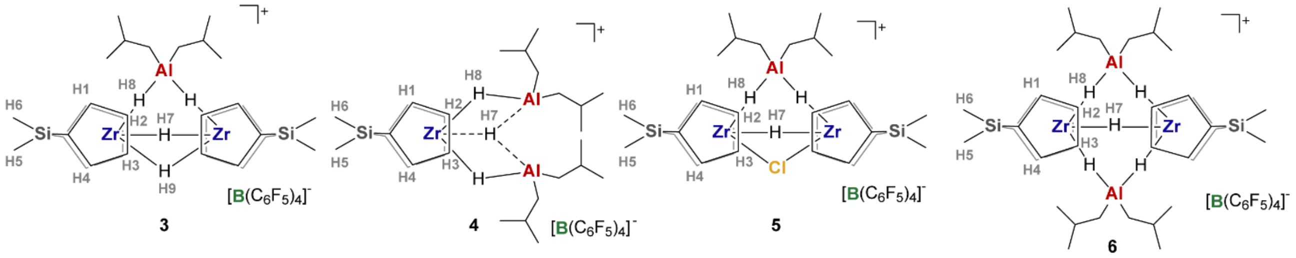

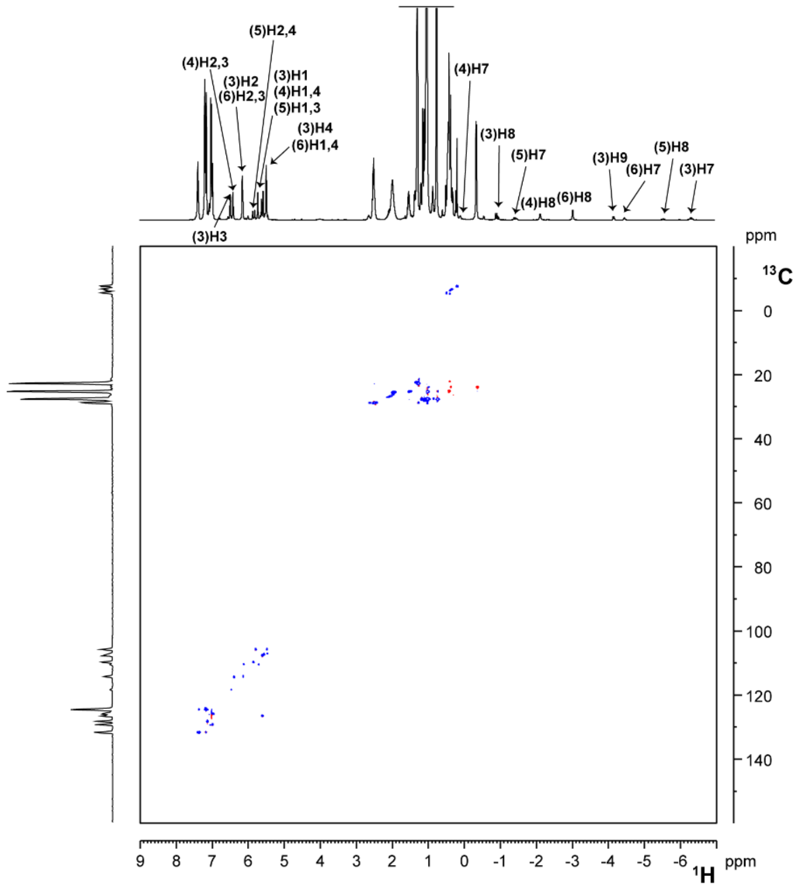

Figure S9. ^1H-^{13}C HSQC NMR spectrum of the products of the reaction between **2_NHC** and DIBAL-H at 298 K in chlorobenzene-d_5.

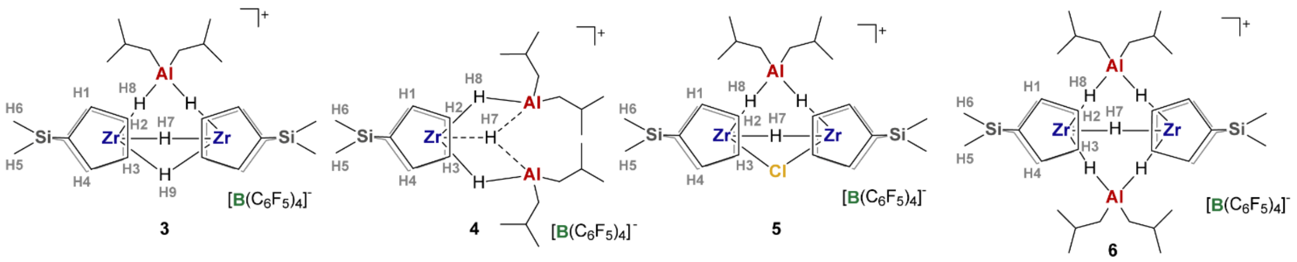

3 **4** **5** **6**

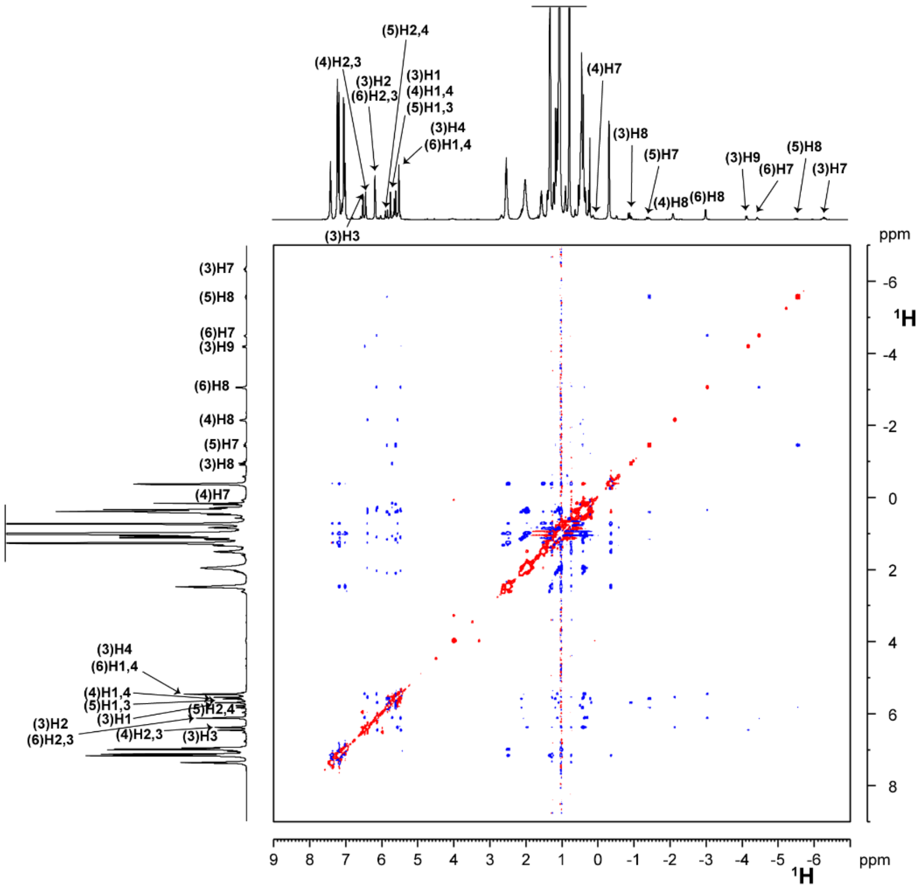

Figure S10. ^{1}H NOESY NMR spectrum of the products of the reaction between **2_NHC** and DIBAL-H at 298 K in chlorobenzene-d_5.

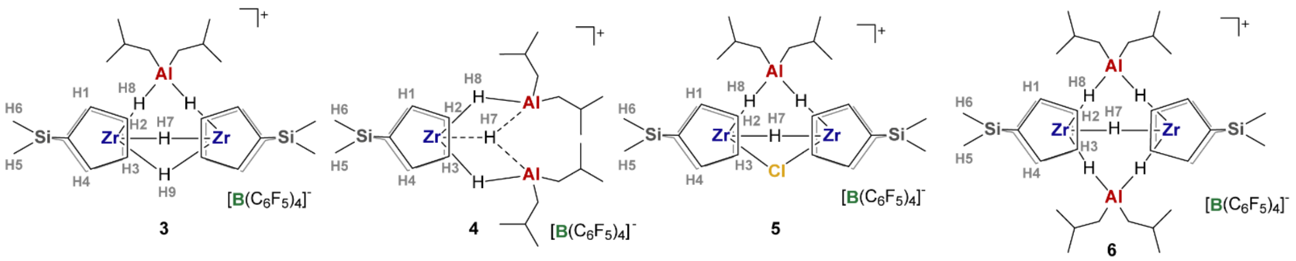

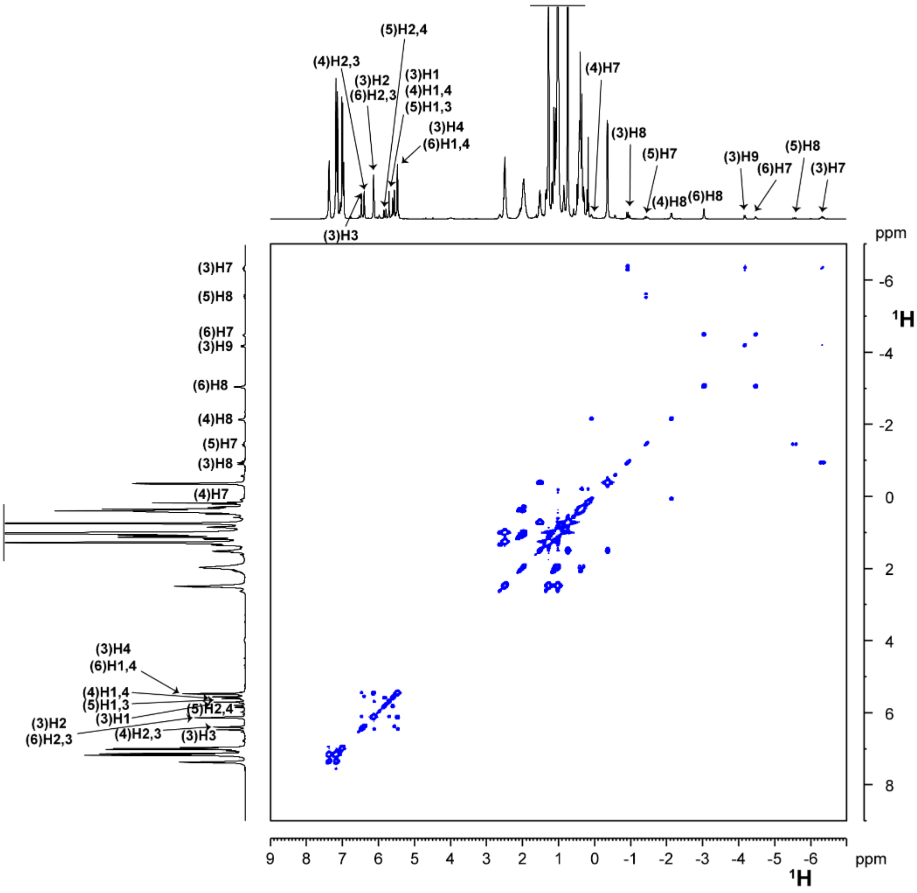

Figure S11. ^{1}H COSY NMR spectrum of the products of the reaction between **2_NHC** and DIBAL-H at 298 K in chlorobenzene-d_5.

1.3 Reaction with AlHAl_DMA

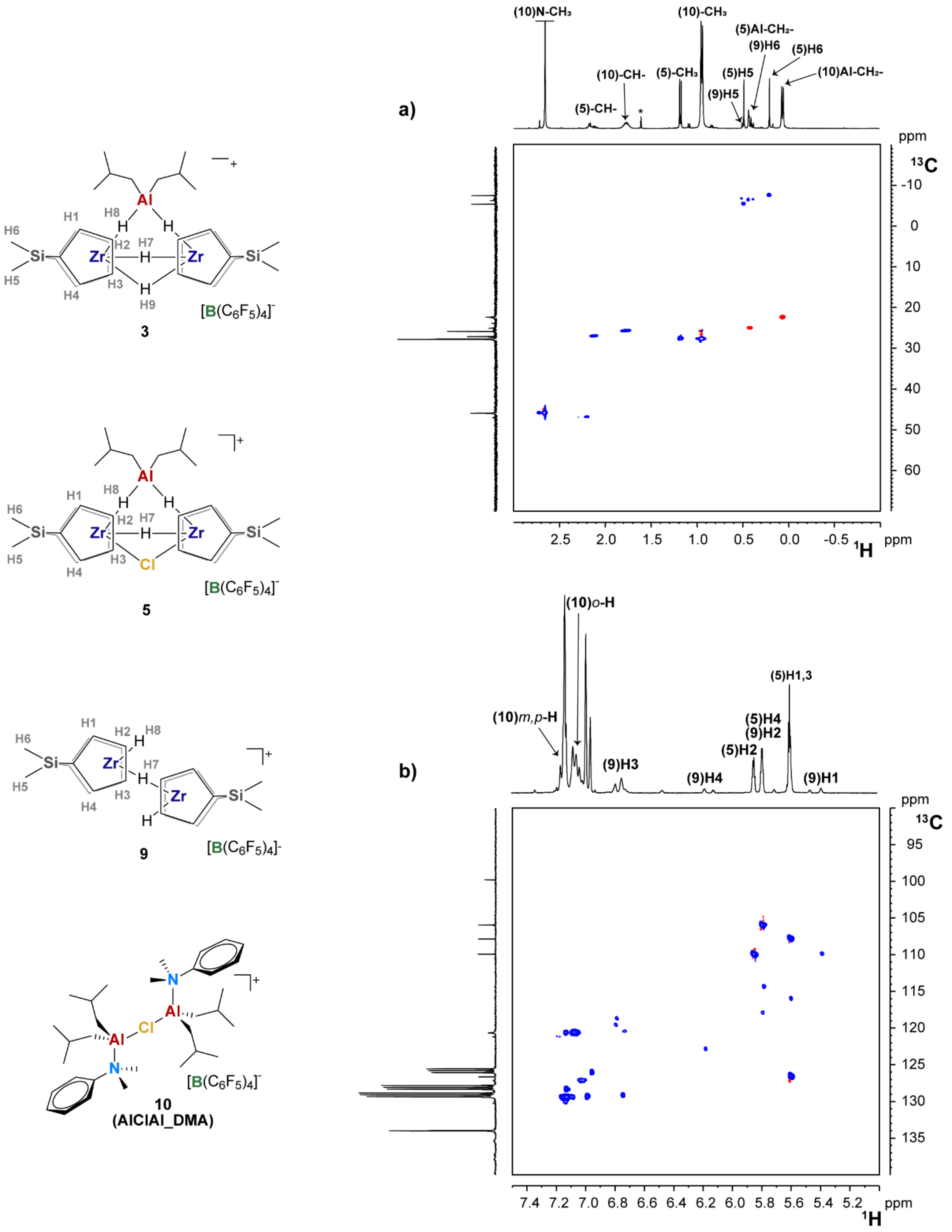

Figure S12. Sections of ^{1}H-^{13}C HSQC NMR spectrum of the products of the reaction between (Me_2SiCp_2)$ZrCl_2$ and **AlHAl_DMA** at 298 K in chlorobenzene-d_5.

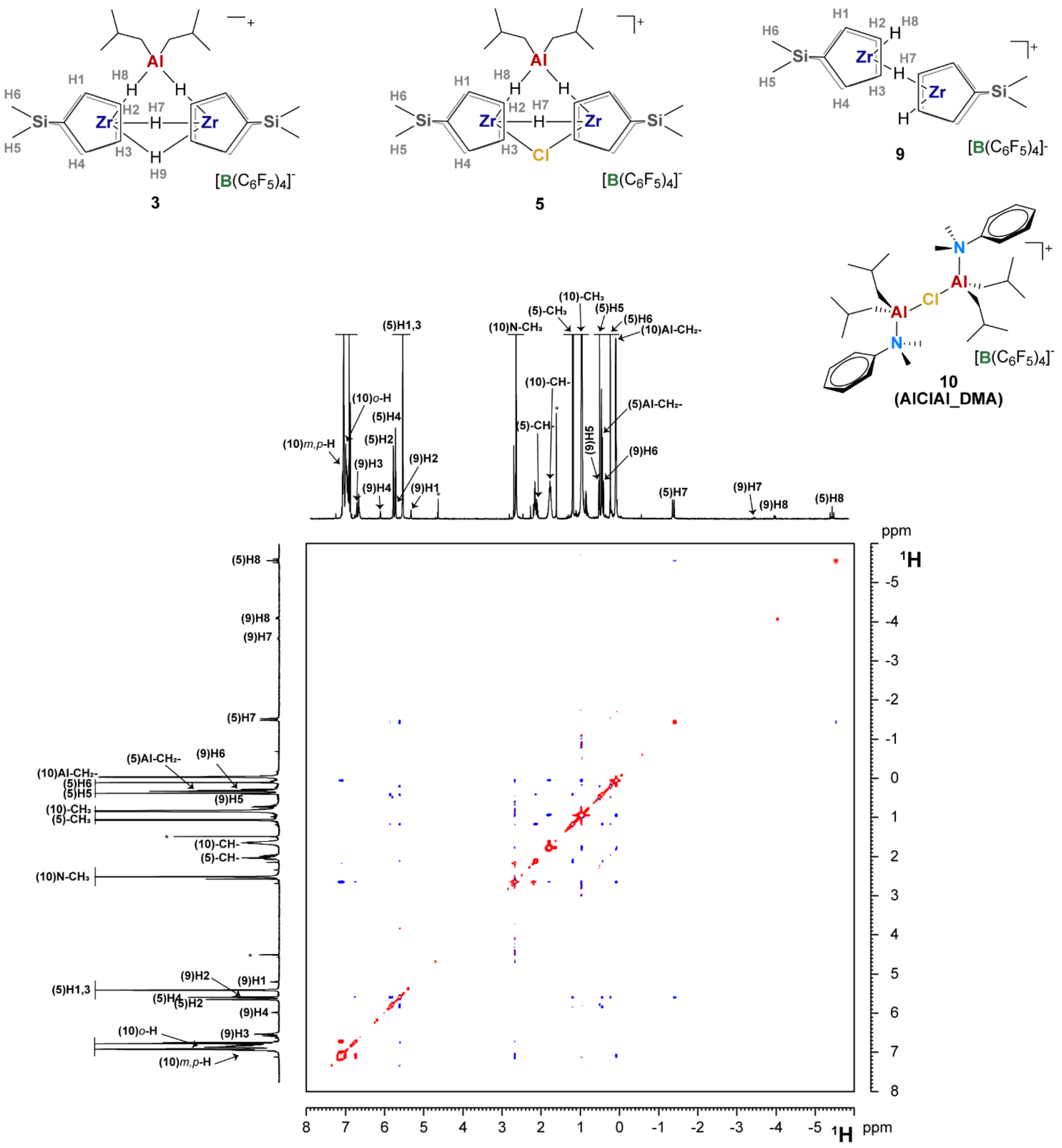

Figure S13. ^{1}H NOESY NMR spectrum of the products of the reaction between $(Me_2SiCp_2)ZrCl_2$ and **AlHAl_DMA** at 298 K in chlorobenzene-d_5.

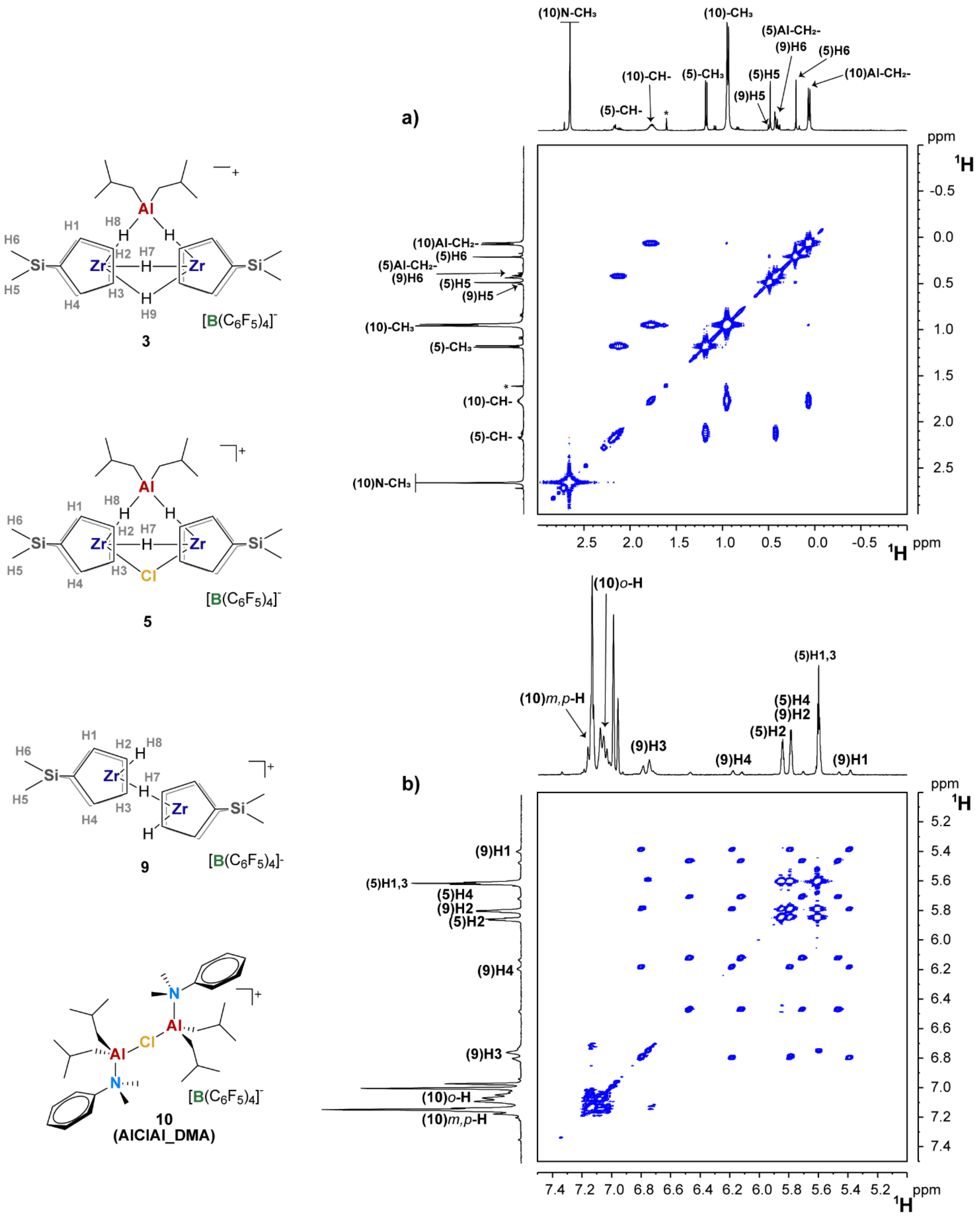

Figure S14. Sections of ^{1}H COSY NMR spectrum of the products of the reaction between (Me_2SiCp_2)$ZrCl_2$ and **AlHAl_DMA** at 298 K in chlorobenzene-d_5.

3.4 Scavenging ability of AlHAl_DMA

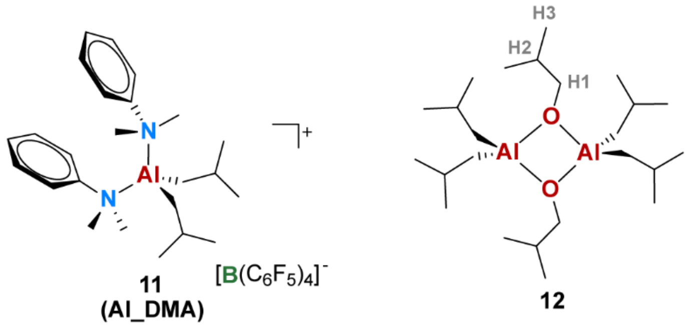

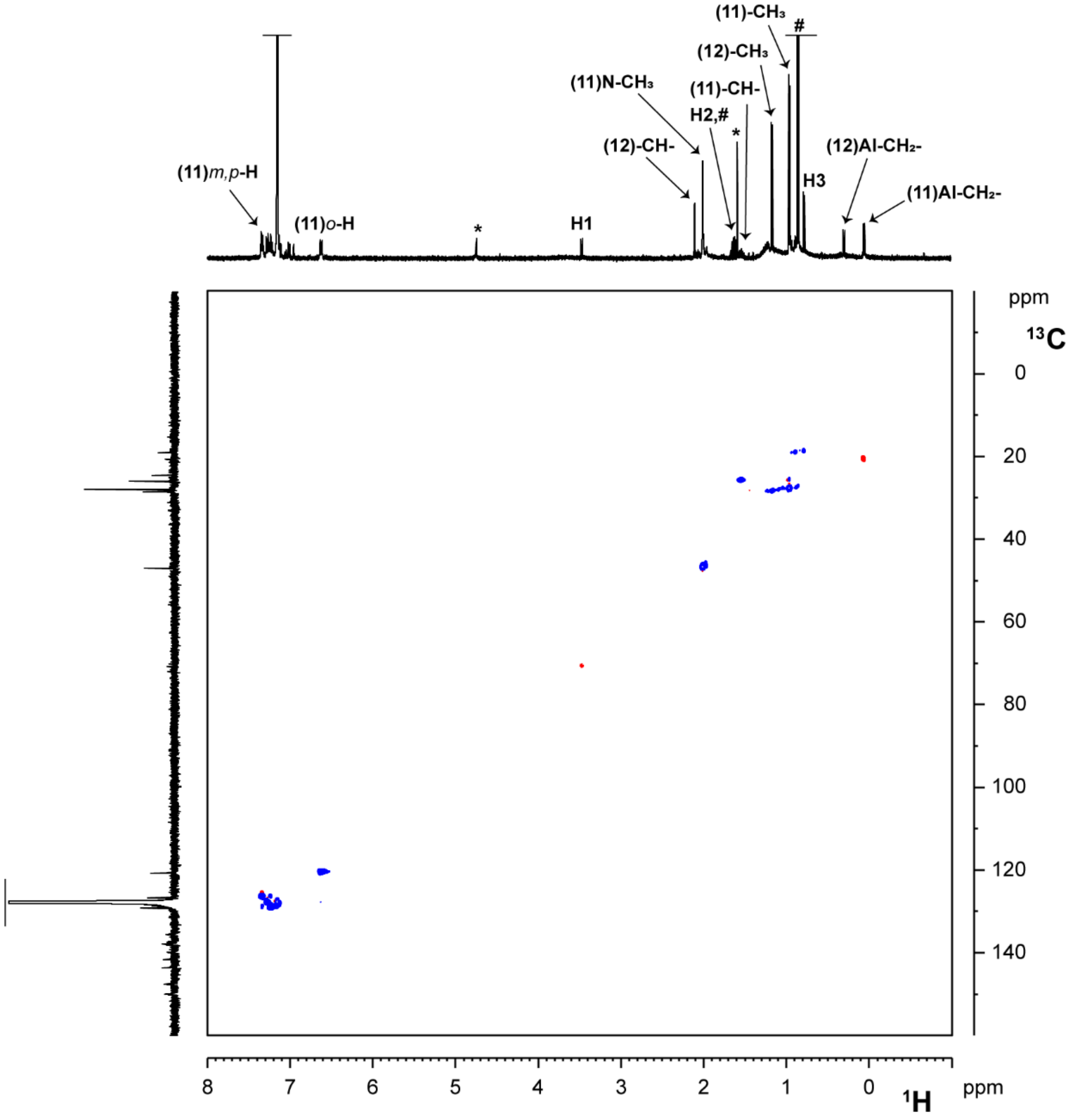

Figure S15. ^{1}H-^{13}C HSQC NMR spectrum of the products of the reaction of **AlHAl_DMA** with atmospheric oxygen and moisture at 298 K in benzene-d_6.

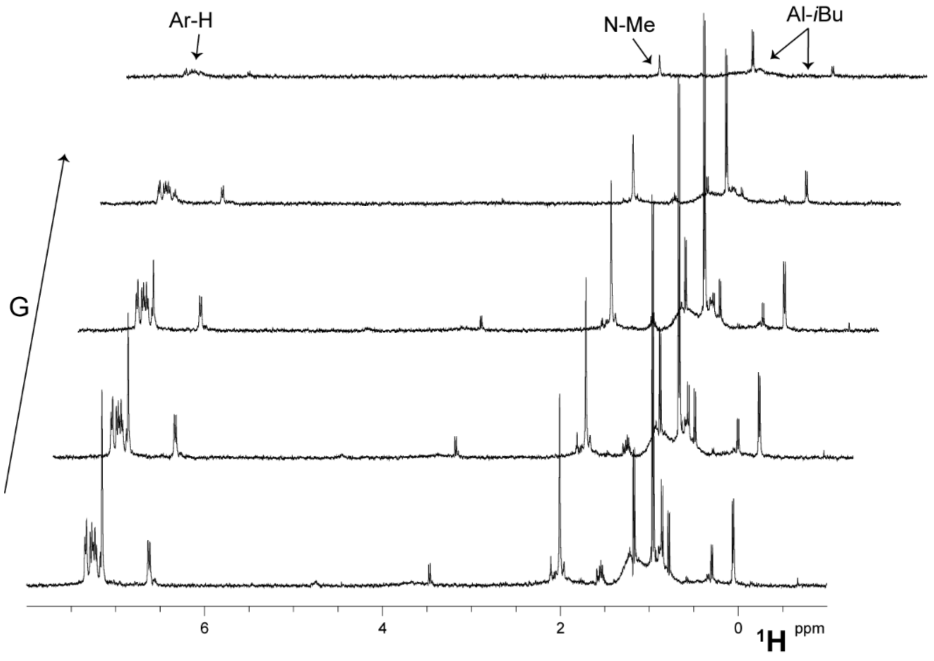

Figure S16. ^{1}H PGSE NMR spectra at increasing gradient strength (G) of the products of the reaction of **AlHAl_DMA** with atmospheric oxygen and moisture at 298 K in benzene-d_6. The broad signals suggesting the presence of DMA-decorated *iso*-butylaluminoxanes are highlighted.

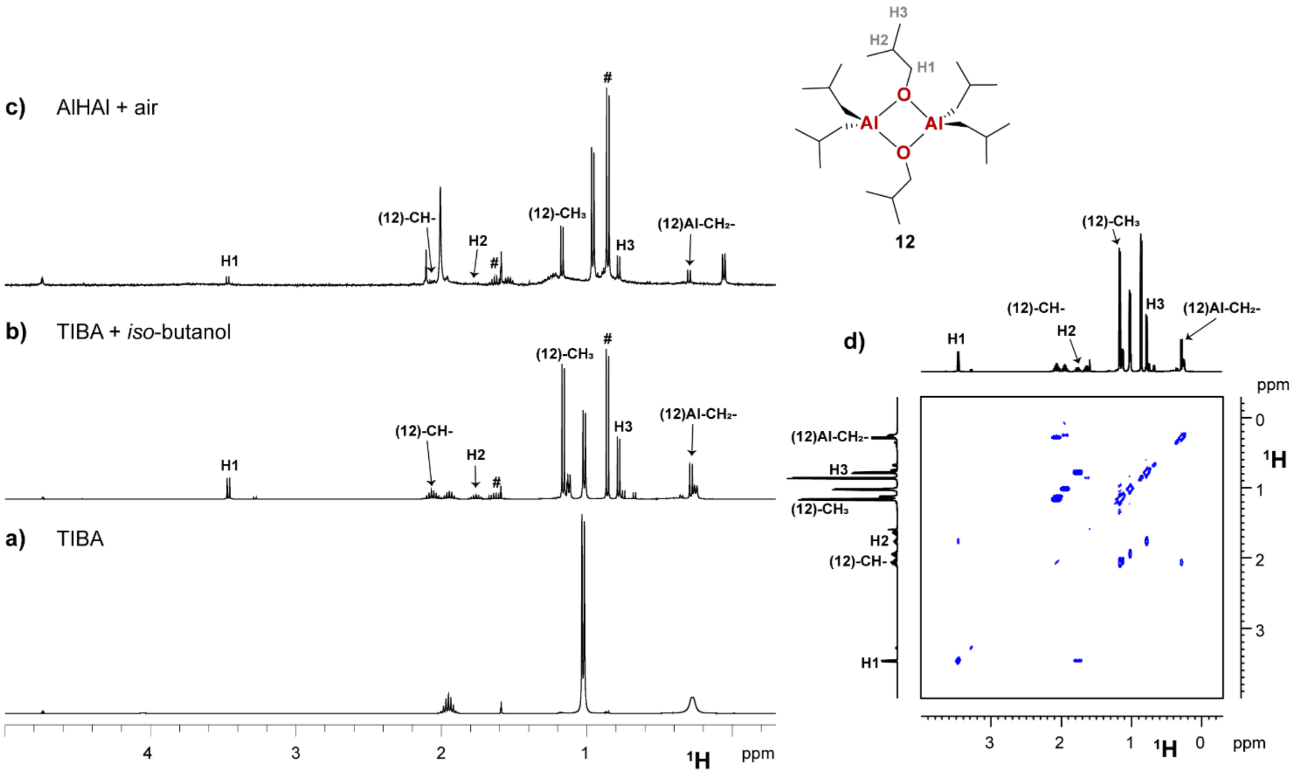

Figure S17. Comparison of ^{1}H NMR spectra (benzene-d_6, 298 K) of a) TIBA, b) crude reaction mixture of TIBA and *iso*-butanol and c) the reaction mixture obtained by reacting **AlHAl_DMA** with air after 3 days (same as in Figure 6a), and d) ^{1}H COSY NMR spectrum of the crude reaction mixture of TIBA and *iso*-butanol. The signals of **12** are highlighted. # = *iso*-butane.

2. X-ray crystallographic details

Table S1. Crystallographic details for **7**.

**Complex**	**7**
**CCDC n.**	**2466602**
Elemental Formula	C_{48} H_{28} B Cl_3 F_{20} Zr_2 Si_2
Formula weight	1340.48
Crystal system	Monoclinic
Space group	Pn
Unit Cell dimensions:	
a = (Å)	10.8438(5)
b =	17.5035(7)
c =	13.2873(6)
α = (°)	90
β =	99.517(2)
γ =	90
Volume ($Å^3$)	2487.28(19)
Z, Calculated density (g/cm^3)	2, 1.790
F(000)	1320
Absorption coefficient (mm^{-1})	0.740
Temperature (K)	150.(2)
Crystal colour, shape	Yellow, Prism
Crystal size (mm)	0.180 x 0.100 x 0.040
On the diffractometer:	
Theta range for data collection	2.5334 to 28.2854
Limiting indices	-14<=h<=14, -23<=k<=23, -17<=l<=17
Completeness	99.5 %
Max. and min. transmission	0.97 and 0.85
Reflns collected (not incl. absences)	41808
No. of unique reflns, R(int) for equivs	12206, 0.0266
No. of 'observed' reflns ($I > 2\sigma_I$)	11708
Refinement:	
Data/restraints/parameters	12206/2/691
Goodness-of-fit on F^2	1.071
Final R indices ('obsd' data)	0.0240, 0.0509
Final R indices (all data)	0.0261, 0.0527
Reflns weighted: 1/w = [a]	\s^2^(Fo^2^) +(0.0228P)^2^+0.4077P
Largest diff. peak and hole (e. $Å^{-3}$)	0.290 and -0.416

[a]where P=(Fo^2^+2Fc^2^)/3

Table S2. Crystallographic details for **8**.

**Complex**	**8**
**CCDC n.**	**2466601**
Elemental Formula	$C_{72}H_{28}B_2F_{40}Cl_2Si_2Zr_2$ + 2 C_6H_5Cl
Formula weight	1104.59
Crystal system	Triclinic
Space group	P -1
Unit Cell dimensions:	
a = (Å)	11.1255(5)
b =	13.4353(6)
c =	14.6793(7)
α = (°)	108.817(2)
β =	97.227(2)
γ =	98.209(2)
Volume ($Å^3$)	2020.75(16)
Z, Calculated density (g/cm^3)	2, 1.815
F(000)	1088
Absorption coefficient (mm^{-1})	0.561
Temperature (K)	145.(2)
Crystal colour, shape	Orange, Prism
Crystal size (mm)	0.300 x 0.100 x 0.100
On the diffractometer:	
Theta range for data collection	2.5634 to 28.2765
Limiting indices	-14<=h<=14, -17<=k<=15, -19<=l<=19
Completeness	99.5 %
Max. and min. transmission	0.7457 and 0.6934
Reflns collected (not incl. absences)	30431
No. of unique reflns, R(int) for equivs	9976, 0.0269
No. of 'observed' reflns ($I > 2\sigma_I$)	8519
Refinement:	
Data/restraints/parameters	9976/365/670
Goodness-of-fit on F^2	1.052
Final R indices ('obsd' data)	0.0399, 0.0951
Final R indices (all data)	0.0492, 0.1017
Reflns weighted: 1/w = [a]	\s^2^(Fo^2^) +(0.0379P)^2^+2.6551P
Largest diff. peak and hole (e. $Å^{-3}$)	1.018 and -0.730

[a]where P=(Fo^2^+2Fc^2^)/3

Table S3. Crystallographic details for **5**.

**Complex**	**5**
**CCDC n.**	**2466603**
Elemental Formula	$C_{56} H_{49} Al B F_{20} Cl_1 Si_2 Zr_2$ + 0.5 $C_6 H_5 Cl$
Formula weight	1470.37
Crystal system	Monoclinic
Space group	P 2(1)/n
Unit Cell dimensions:	
a = (Å)	13.2904(7)
b =	15.5335(8)
c =	29.3592(16)
α = (°)	90
β =	102.145(2)
γ =	90
Volume (Å^3)	5925.4(5)
Z, Calculated density (g/cm^3)	4, 1.648
F(000)	2949
Absorption coefficient (mm^{-1})	0. 578
Temperature (K)	146.(2)
Crystal colour, shape	Green, Cube
Crystal size (mm)	0.200 x 0.160 x 0.080
On the diffractometer:	
Theta range for data collection	2.27 to 28.30
Limiting indices	-17<=h<=17, -20<=k<=20, -39<=l<=39
Completeness	99.6 %
Max. and min. transmission	0.7457 and 0.6667
Reflns collected (not incl. absences)	195300
No. of unique reflns, R(int) for equivs	14679, 0.1309
No. of 'observed' reflns ($I > 2\sigma_I$)	10347
Refinement:	
Data/restraints/parameters	14679/162/808
Goodness-of-fit on F^2	1.111
Final R indices ('obsd' data)	0.0936, 0.1609
Final R indices (all data)	0.1411, 0.1866
Reflns weighted: 1/w = [a]	\s^2^(Fo^2^) +(0.0042P)^2^+65.1061P
Largest diff. peak and hole (e. Å^{-3})	1.265 and -1.340

[a]where P=(Fo^2^+2Fc^2^)/3

The crystal structure of **5** is characterized by high values of R_1, wR_2 and R_{int} due to poor-quality crystals (very weak reflections at resolutions lower than 1.00 Å). Recrystallization attempts were unsuccessful. Solution with unit cells with different symmetries and twinning refinement did not yield results.
